# Personality and city culture predict attitudes and practices toward mosquitoes and mosquito-borne diseases in South Texas

**DOI:** 10.3389/fpubh.2022.919780

**Published:** 2022-11-07

**Authors:** Amy K. Bohmann, Lisset Martinez-Berman, Amy R. Senger, Megan R. Wise de Valdez

**Affiliations:** Department of Life Sciences, Texas A&M University-San Antonio, One University Way, San Antonio, TX, United States

**Keywords:** KAP survey, Big Five personality, health behaviors, urban mosquito ecology, premise condition index, Hispanic majority population

## Abstract

Personality is known to affect compliance with health-protective behaviors and it has been shown that effective public health messaging can be informed by an understanding of that relationship. Thus, we aimed to evaluate the role personality might play in implementing personal protective measures (PPMs) that can prevent mosquito-borne diseases. This is the first mosquito-related knowledge, attitudes, and practices (KAP) study to incorporate a measure of personality using the Big Five: openness, conscientiousness, extraversion, agreeableness, and neuroticism. KAP studies in Gulf-coast and Mexican border-states in the U.S. are few. Ours is only the second KAP study to take place in Texas despite known local transmission and established mosquito populations capable of transmitting dengue, zika, chikungunya, and West Nile viruses. The KAP survey was administered in three neighborhoods in San Antonio, a large, Hispanic-majority, urban city that is segregated economically and ecologically. We found that openness, agreeableness, and extraversion predicted certain attitudes and PPMs, and that KAP and personality measures did not differ along ethnic or neighborhood lines. Perceptions toward the city's role in mosquito control and education was an important factor in predicting PPMs, suggesting that city culture (attitudes common throughout the city as opposed to attitudes differing by ethnicity and neighborhood) may be most salient in developing public health messaging in San Antonio.

## Introduction

Public health messaging should be tailored to individual communities in order to maximize compliance with health protective behaviors and thereby increase the success of initiatives aimed at preventing the spread of mosquito-borne diseases (MBDs). Knowledge, attitudes, and practices (KAP) surveys are a standard tool in helping to gather information about communities to help tailor that messaging (1).

In addition to differences among communities, mosquito abundance and MBD risk varies geographically (2, 3), further highlighting the fact that knowledge, attitudes, and practices concerning MBDs are not the same in every community. While multiple KAP surveys for mosquitoes and MBDs have been carried out in the United States, most have been conducted in the Northeastern U.S., Eastern coastal states, and Colorado, where West Nile virus (WNV) is the primary MBD present (4–6). These KAP studies represent the KAP of only a fraction of the diverse population of the U.S. and focus on only one MBD. Only four KAP studies have assessed communities in Gulf Coast and Mexican Border States where other mosquito-borne viruses, such as dengue viruses (DENV), zika virus (ZIKV), and chikungunya virus (CHIKV), have been reported or have the potential to establish (7–10). In addition to the difference in MBD risk of these areas compared to the Northern States, the populations of people living in the Gulf Coast and Mexican Border States are ethnically, educationally, and economically different from those areas and each other. In Texas, only one mosquito-related KAP study has been conducted (10) despite the fact that WNV occurs yearly across the state and local transmission of ZIKV, DENV, and CHIKV has occurred along the border (11). This is also surprising because Texas is the second largest State geographically and has three of the 10 most populated cities in the U.S.; Houston, San Antonio, and Dallas. All three cities are primary travel hubs of people coming in from Central America where ZIKV, DENV, and CHIKV are endemic. Because these cities harbor the species of mosquitoes capable of transmitting ZIKV, DENV, and CHIKV and because global warming may lead to northern expansion of these tropical MBDs, Texas communities are considered at risk (12). The one KAP study in Texas was conducted in several small communities along the Texas-Mexico border (10) and therefore cannot represent the KAP of the larger and more diverse communities of the urban metropolises at risk in Texas. Thus, there is a need for KAP studies in these areas.

In order for public health messaging to be effective, we have taken a collaborative and inter-disciplinary approach to our KAP study by including assessment tools and analyses used by social psychologists (13, 14) to evaluate attitudes and predict health behaviors. Our intent is to show that psychological factors may play a role in the effectiveness of messaging people receive. KAP studies include an attitudinal component, which is studied extensively in social psychology, and we have chosen to properly include that component by creating a scale that accurately measures attitudes. With the recent COVID-19 pandemic, the value of this type of collaboration has become abundantly clear as we strive to find ways to increase community-wide compliance with preventative practices. The new collaborative approaches we have implemented in this study include a robust attitude measure and a measure of the Big Five personality traits (15).

Personality is a factor that can affect compliance with health-related behaviors (16) and which had not been incorporated into KAP studies. Personality is viewed as an enduring and consistent characteristic that plays a role in shaping the intention of actually doing a particular health-related behavior (17, 18). Personality may also influence whether one perceives a situation as having risk or a potentially negative outcome, further influencing one's behavior toward that target (19). The personality model used for the current study was the Big Five personality model (15). The Big Five model of personality includes the following traits: Openness, Conscientiousness, Extraversion, Agreeableness, and Neuroticism. Bermúdez (16) reviewed correlative evidence that some personality traits may predispose individuals to practice behaviors that are beneficial to their health while other personality traits may contribute to poor health behaviors. Conscientiousness, a trait which encompasses timeliness, orderliness, and structure, was of interest because it has a history of positively correlating with beneficial health behaviors (20, 21) and negatively correlating with detrimental health behaviors, such as risk-taking (22). Openness, a trait which means a person is open to new experiences and ideas, was of interest because it has been shown to be positively associated with adherence to health guidelines (23, 24). Also, both conscientiousness and neuroticism have been positively correlated with health behaviors related to cancer treatment (25), and both conscientiousness and openness predicted adherence to COVID-19 preventative measures (24, 26). Agreeableness also positively correlated with compliance behaviors in preventing COVID-19 and this personality trait was more prevalent in women (27). Nudelman and Ivanova (28) also concurred that conscientiousness predicted a variety of health behaviors including, eating regular meals, sleeping at least 7 h a night, and protecting oneself from the sun. Extraversion was negatively correlated with beneficial health behaviors (29, 30). Understanding which personality traits are associated with health-positive behaviors, especially those preventing community-based health risks, can assist public health officials with appropriate messaging aimed at increasing community-wide compliance (19, 31, 32). There have been no studies which have assessed whether personality traits may play a role in compliance with practices aimed at preventing mosquitoes and therefore, a potential avenue for improving and tailoring public health messaging has been left unexplored.

Sheeran et al. (33) found support for interventions that modify attitudes that promote behavior change in a health-related context. Social psychologists define attitudes as positive or negative evaluations of a target (ex. mosquitoes or MBDs), which include emotional, behavioral and cognitive components (34, 35). Thus, it is important to accurately evaluate attitudes for appropriate target messaging. In social psychology this is done by conducting a primary study to create a comprehensive attitude scale through a factor analysis before deploying the scale in the target community. A factor analysis more accurately identifies attitudes that can be assessed by multiple items; it is recommended to have at least three items per attitude for good reliability of the scale (36). Attitudes measured with only one item, as is the norm in most mosquito-related KAP studies, do not share this reliability advantage (37, 38).

In this study we aimed to fill the gaps in our knowledge of how communities perceive mosquitoes and MBDs by (1) including a robust measure of attitudes to more accurately evaluate attitudes toward mosquitoes and MBDs, (2) developing and using a Knowledge, Attitude, *Personality*, and Practice (KAPP) survey to explore how personality might be used in future mosquito KAP studies, and (3) by conducting only the second mosquito KAP study in Texas and the first to be deployed in a large, economically and ecologically diverse, urban U.S. city (>1 million) with a Hispanic-majority.

## Materials and methods

### Study 1–attitude scale

In order to ensure statements on our KAPP survey (Study 2) were measuring attitudes appropriately, an attitude scale was created.

#### Participants

We recruited, without compensation, participants from the Texas A&M-San Antonio University participant pool, and for the purpose of age diversity, we also recruited from social media contacts and word of mouth. In all, 180 participants accessed the survey from a Qualtrics © (2018) link. Males comprised 33% of the sample, females 65%, and 2% indicated “other.” The sample included 59% Hispanic participants, 32% White, 3% Black, and 6% Other, with a mean age of 34.04 (SD = 13.63).

#### Procedure

We generated 69 statements regarding various attitudes toward mosquitoes and MBDs. These items were answered by participants on a five-point Likert-type scale in Qualtrics© (2018). The attitude items were principle component factor analyzed [SPSS 27.0. Armonk, NY: IBM Corp; (39)] into a six-factor model containing 29 of the original 69 items that were parsed out into six different attitudes each with multiple statements to assess them (Table 1); Mosquitoes are a risk (6 statements), Mosquito diseases are serious (4 statements), Fear of mosquito-borne diseases (3 statements), Fear of mosquitoes (4 statements), The role the city plays in mosquito control and education is sufficient (4 statements), Yard maintenance is important (7 statements; Table 1). Attitude factors had eigenvalues between 1.89 and 5.6. Our eigenvalues indicate that the variance explained by each factor is reasonable (38). Additionally, all attitude factors had a Cronbach's alpha reliability score of at least 0.74, with most above 0.86; these values are considered an acceptable measure of how closely the items within each attitude are related (41). This scale was used as the attitude portion in the KAPP of Study 2.

**Table 1:** Parameters used to calculate Premise Condition Index [modified from Tun-Lin et al. (40)].

	**Score**		
	**1**	**2**	**3**
**Parameter assessed**
House condition	House in good repair	House with little-some damage	House in poor repair
Yard condition	Tidy yard and landscaping maintained	Lawn cut but not watered, or some overgrowth of landscaping	Untidy yard, no maintenance of landscaping, trash present
Gutter condition	No gutters	Present but in good repair	Present with visible clogging
Amount of shade	<25%	>25% but <50%	>50%
Container frequency	0–4	5–10	11+

Maximum PCI Score = 15, Minimum PCI Score = 5. The higher the score the more suitable the habitat for larval mosquitoes.

### Study 2-KAPP

#### Study site

San Antonio, TX is the 7^th^ largest city in the U.S. with a population of over 1.4 million, a Hispanic majority of 63.9% (42) and is located ~100 miles from the Mexican border. Mosquito surveillance in San Antonio is new. In 2016, San Antonio developed a ZIKV education campaign but it was not until 2019 that the San Antonio Metropolitan Health District (SAMHD) initiated a formal mosquito surveillance program. San Antonio is considered one of the most economically segregated cities in the U.S. (43) and this is reflected ecologically as well. Affluent areas are located in the north which is dominated by hill country with old growth live-oak trees, whereas the lower-income areas are in the south and are dominated by Blackland prairie and South Texas shrub land (44). Previous studies in San Antonio have shown that there is potential for differences in mosquito populations based on the socioeconomic status and ecology of the neighborhoods (45, 46). Factors associated with SES of neighborhood, like differences in how one cares for their yard (excessive or absence of watering, removal or allowing of overgrowth, availability of funds for mosquito control), as well as ecological differences are important to incorporate into mosquito-related public health messaging designed to reduce MBD risk in the U.S. (2, 8, 10, 47).

**Figure 1:**
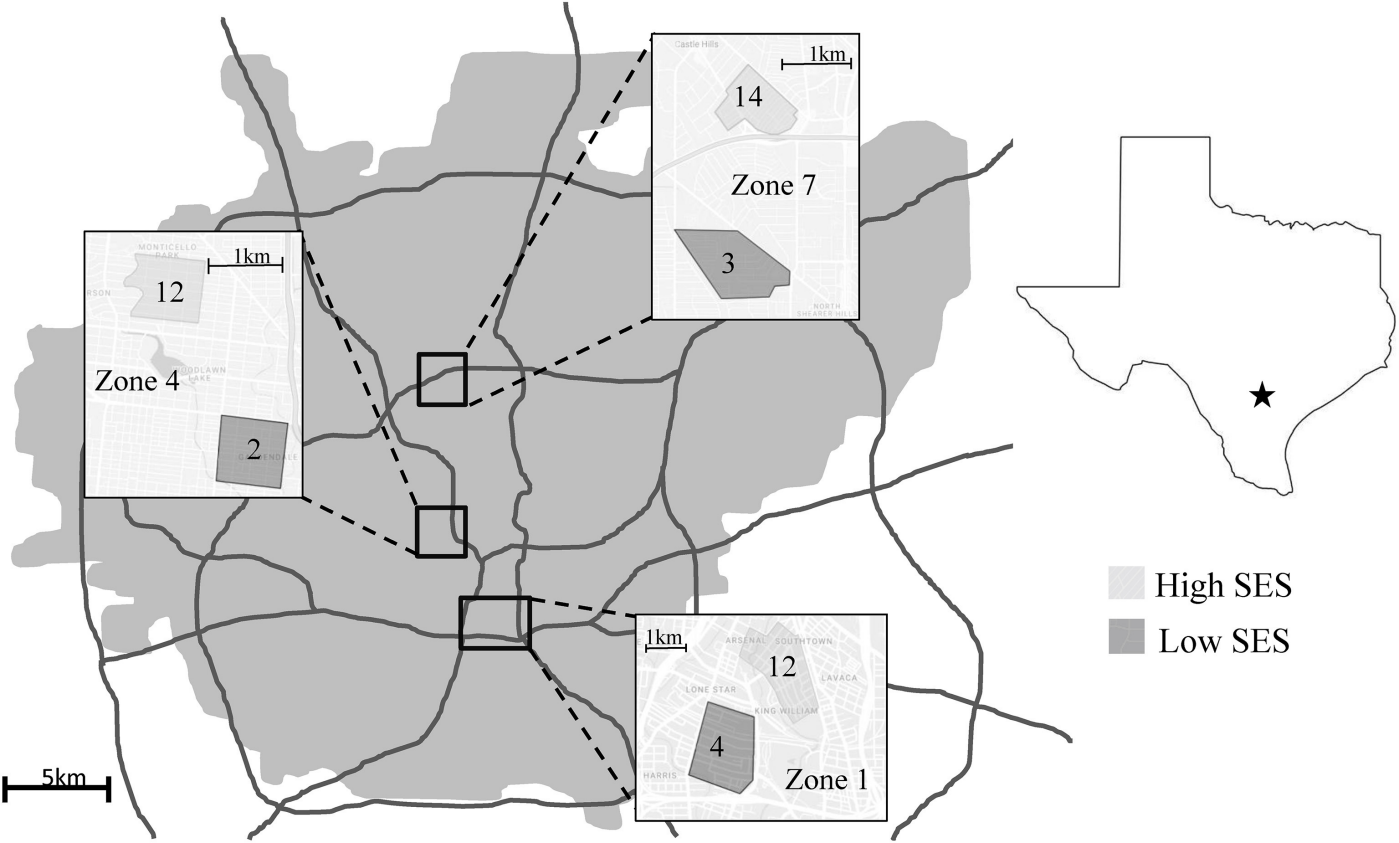
Study site locations. Shaded boxes are the selected neighborhoods of different SES. Numbers inside the boxes are number of completed surveys collected. Note that three respondents are not included in this figure due to not listing their address on the on-line survey. Figure modified from (45, 46).

Three areas in San Antonio previously identified as having at least moderate levels of mosquito activity, having both high and low-income neighborhoods within each, and which are part of on-going mosquito-related studies (46) were selected for questionnaire distribution; Zones 1, 4, and 7 [(46, 48); Figure 1]. These areas represent an urban center neighborhood (Zone 1, homes built beginning in the late 1800's), a peri-urban neighborhood (Zone 4, homes built beginning in the 1920's), and a centralized well-established suburban neighborhood (Zone 7, homes built beginning in the 1950's). Within each area, we selected two neighborhoods that were visibly different in socio-economic status (SES) in order to improve our chances of obtaining a socioeconomically diverse pool of respondents. We used an informal visual evaluation of the yards and home maintenance to make an assessment of whether the neighborhood was low- or high-SES [(48); Figure 1].

#### Participants

The first week of July, 2018, we sent letters asking for participation in a KAPP survey to 108 residents who previously agreed to allow mosquito collections on their property as part of prior mosquito studies (46) with the aim to evaluate their KAPP responses with mosquito population data. We also went door-to-door and visited a similar number of homes in each neighborhood within each zone (77–95 homes in each, total = 261 homes) from July 16 to July 30, 2018 to increase the number of survey participants. Homes in a single neighborhood were visited sequentially by address between the hours of 9:00 am−12:00 pm. If a resident answered the door, agreed to take the survey, and completed it, we included their survey in our study. If the residents were not home, a copy of the letter with a link to the survey was left at the home to encourage online participation. In total 469 residents were contacted (108 letters and 261 homes visited) and 50 people completed the survey, a response rate of 10.6%.

#### Premise condition index

A premise condition index (PCI) is a numerical score calculated by mosquito control personnel to quickly assess the larval habitat suitability of a home in order to determine whether the home should be targeted for mosquito control measures (40). A low PCI indicates low habitat suitability and a high PCI indicates high habitat suitability [(40); Table 2]. We trained several student researchers to use the PCI to assess the homes of each participant. Because several personal protective measures (PPMs) are associated with yard and home maintenance a PCI can be another way to evaluate the success of these practices.

**Table 2:** Attitude scale factor analysis and reliabilities.

**Attitude factor and questions clustered**	**Cronbach's α^a^**	**Eigenvalue^b^**
**Mosquitoes are a risk**	0.78	1.830
1. I would rather stay indoors than go outside without mosquito repellent. 2. I am motivated to put on mosquito repellent 3. It makes me nervous if I forget to put on mosquito repellent 4. There is a mosquito problem in my neighborhood 5. There is a mosquito problem at my house 6. I worry about mosquitoes every time I am outside		
**Mosquito diseases are serious**	0.92	3.266
1. Zika is a serious disease 2. Dengue fever is a serious disease 3. West Nile is a serious disease 4. Mosquitoes can transmit diseases		
**Fear of mosquito-borne diseases**	0.98	2.053
1. I am afraid of getting West Nile virus 2. I am afraid of getting the Zika virus 3. I am afraid of getting dengue fever		
**Fear of mosquitoes**	0.88	5.627
1. I am afraid of insects 2. I am afraid of mosquitoes 3. I fear getting bitten by mosquitoes 4. I fear getting bitten by insects		
**The role the city plays in mosquito control and education is sufficient**	0.86	3.842
1. The city of provides information about mosquitoes 2. The city of provides enough information about West Nile Virus 3. The city of provides enough information about dengue Fever 4. The city of San Antonio provides enough information about Zika 5. The city of San Antonio does a good job of controlling and preventing mosquitoes		
**Yard maintenance is important**	0.74	3.014
1. Yard appearance is important to me 2. I have sufficient time for yard maintenance 3. I have sufficient funds for yard maintenance 4. I need to have a clean yard 5. I care about what others think about my lawn 6. I have sufficient funds for mosquito repellent 7. Mosquito control is important to me		

a Cronbach's a is an inter-item reliability measure.

b Eigenvalues indicate amount of variance accounted for by that factor in a factor analysis; values > 1 are considered good.

#### KAPP questionnaire

The knowledge portion of the survey was adapted from questions used by Tuiten et al. (49) and the World Health Organization (1), and included not only questions about WNV, but also ZIKV and DENV (Survey is in Supplementary materials). Participants were assigned five knowledge scores based on correct answers to questions about mosquitoes in general (6 questions), WNV (8 questions), ZIKV (6 questions), and DENV (6 questions). The fifth knowledge score was a sum total of answers to all knowledge-based questions.

Participants indicated the frequency with which they employ the following PPMs: wearing protective clothing when outside, eliminating standing water, using insect repellant, using insecticides, and using *Bacillus thurengiensis israelensis* (BTI) briquettes/pellets. In addition to frequency, we later dichotomized the likert-based responses into yes/no answers in terms of whether the frequency was considered preventative against mosquitoes or not (Supplementary Figure 1). For example, participants were originally asked at what frequency they eliminated standing water: never, once a month, twice a month, or once a week. The only response that indicated appropriate use of the practice was “once a week.” Thus, those responses scored a “yes” and all others a “no.” For other practices, the options were never, occasionally, half the time, often, always, or don't know. Responses of often or always were “yes,” responses of never, occasionally, or half the time were “no.” Responses of “didn't know” were not used.

To measure personality, we used the well-established Mini International Personality Item Pool [Mini-IPIP; (50)], which is a 20-statement measure of the Big Five personality factors: Openness, Conscientiousness, Extraversion, Agreeableness, and Neuroticism (15). Agreement with the statements in each personality factor indicated a higher score on that factor. Participants receive an average score on each personality factor, the higher the average score, the more they identified with that particular personality. The participants' scores on each personality were used in correlational and multiple regression analyses to evaluate the role of personality in practicing PPMs related to mosquitoes.

#### Data analysis

We conducted a Pearson correlation analyses of the attitude scale factors, personality factors, knowledge, frequency of practices, demographics, and PCI. We also conducted independent *t*-tests to evaluate the relationships between the dichotomized practices and attitudes, personalities, knowledge, and demographics (SPSS v27.0 2020 Armonk, NY: IBM Corp). In order to evaluate how personality factors, attitude factors, and mean knowledge scores predicted practices, we used two strategies for multiple regression analyses to evaluate these relationships. We constructed global models using only attitudes or only personalities using simultaneous entry to predict practices (SPSS as listed above). We also constructed models with fewer predictors using forced entry (JASP 2021 v0.16, Amsterdam, The Netherlands) in order to alleviate potential issues related to collinearity among independent predictors. When constructing the final models, we intentionally chose models with six or fewer predictors as that was most appropriate for our sample size. We screened individual factors [*p* < 0.25; (47)], and final models (*p* ≤ 0.05) were selected using backward design. We limited the number of subjects per predictive variables to at least five as that can accurately estimate regression coefficients (51, 52).

### Ethical considerations

Human subject approval was provided by A&M-SA Institutional Review Board (protocol # 2018-49).

## Results

### KAPP respondent demographics

Fifty individuals participated in the KAPP survey, 16 from Zone 1, 14 from Zone 4, and 17 from Zone 7 (Figure 1). Three respondents, who accessed the survey on line, did not include their address and thus we were unable to assign them to a zone. While we aimed to collect surveys from neighborhoods of different SES within the zones in order to increase the economic diversity of our respondents, only nine residents in what we presume were low-SES neighborhoods participated Additionally, only 20 participants had mosquito data associated with their residence from our previous studies (46, 48), and therefore we did not include mosquito population data in our analyses.

The participants were majority female, White/non-Hispanic, college-educated, and earned more than $50,000 a year (Table 3). Those that identified as Hispanic represented 35.3% of the participants. Only three individuals reported a race/ethnicity other than white/non-Hispanic or Hispanic (Table 3) and because the number in that category was not sufficient for analyses, we focused on white/non-Hispanic vs. Hispanic ethnicity results only. Comparison of education and income between white/non-Hispanic and Hispanic respondents showed no significant differences (Supplementary Table 1). The average PCI Score was 8.57 (SD 1.70) with a range of 5–13 (Table 3). The PCI, race/ethnicity, age, income, nor education differed among zones (Supplementary Table 2).

**Table 3:** Demographic characteristics of respondents.

**Characteristic**	***n* (%)**
**Gender**
Male	20 (39.2)
Female	28 (54.9)
No response	3 (5.9)
**Age, years**	
18–21	2 (4)
22–37	7 (14)
38–53	11 (22)
>54	24 (48)
No response	6 (12)
**Race/Ethnicity**
White/non-Hispanic	27 (52.9)
Black	1 (2)
Hispanic	18 (35.3)
Other	2 (3.9)
No response	3 (5.9)
**Highest level of education**
High school diploma	2 (3.9)
Some college or 2-year degree	12 (23.5)
Bachelor's degree	9 (17.6)
Master's, law or other graduate degree	8 (15.7)
Ph.D.	3 (5.9)
No response	17 (33.3)
**Annual household income**
<$14,999	2 (3.9)
$15,000–$49,999	11 (21.5)
$50,000–$99,999	12 (23.5)
$100,000–$149,999	9 (17.6)
$150,000–$199,999	5 (9.8)
> $200,000	3 (5.9)
No response	9 (17.6)

### Knowledge

Knowledge of WNV, ZIKV, and DENV was high with 100, 98, and 79% of respondents, respectively, having heard of the diseases, and the majority knew they were transmitted by mosquitoes (Table 4). Additionally, the majority of respondents knew that ZIKV is transmitted congenitally (54.2%). Fewer respondents (46.9%) knew whether the species of mosquitoes that transmitted these diseases were present in their community. Over 97% of respondents understood where mosquitoes breed. Most respondents (75.5%) trusted SAMHD and their physicians for correct information about MBDs (Table 4).

**Table 4:** Knowledge and attitudes of San Antonio community participants as they relate to MBDs.

**Knowledge**	**Response**				**#Pos. response/Total(%)**
WNV knowledge	Has heard of WNV				49/49 (100)
	Knows it is transmitted by mosquitoes				39/49 (79.6)
	Knows the species of mosquitoes is present in their community				23/49 (46.9)
ZIKV knowledge	Has heard of ZIKV				48/49 (98)
	Knows it is transmitted by mosquitoes				43/48 (89.6)
	Knows it is transmitted sexually				14/48 (29.2)
	Knows it is transmitted congenitally				26/48 (54.2)
	Knows the species of mosquitoes is present in their community				26/48 (54.2)
DENV knowledge	Has heard of DENV				39/49 (79.6)
	Knows it is transmitted by mosquitoes				33/39 (67.3)
	Knows the species of mosquitoes is present in their community				12/39 (30.8)
General knowledge	Knows that mosquitoes breed in water-filled containers				46/47 (97.9)
Entity trusted for information	Friends and family				6/49 (12.2)
	Radio				10/49 (20.4)
	Television				19/49 (38.8)
	Internet				21/49 (42.9)
	Newspaper				22/49 (44.9)
	City of San Antonio metro health				37/49 (75.5)
	Their physician				37/49 (75.5)
**Attitudes**	***N***	**Mean** ^*^ **(SD)**	**Median** ^*^	**Mode** ^*^	**95% CI**
Mosquitoes are a risk	50	3 (0.81)	3	3	0.229
MBDs are serious	50	5 (0.55)	5	5	0.156
Fear of mosquitoes	49	2 (1.11)	3	1	0.320
Fear of MBDs	50	3 (1.53)	3	1	0.434
Role of city is sufficient	50	3 (1.13)	3	3	0.321
Yard maintenance is important	50	4 (0.61)	4	4	0.174

* Likert scale: 1 = strongly disagree, 2 = somewhat disagree, 3 = neutral, 4 = somewhat agree, 5 = strongly agree. Responses were rounded to the nearest whole number for ease in interpretations.

**Figure 2:**
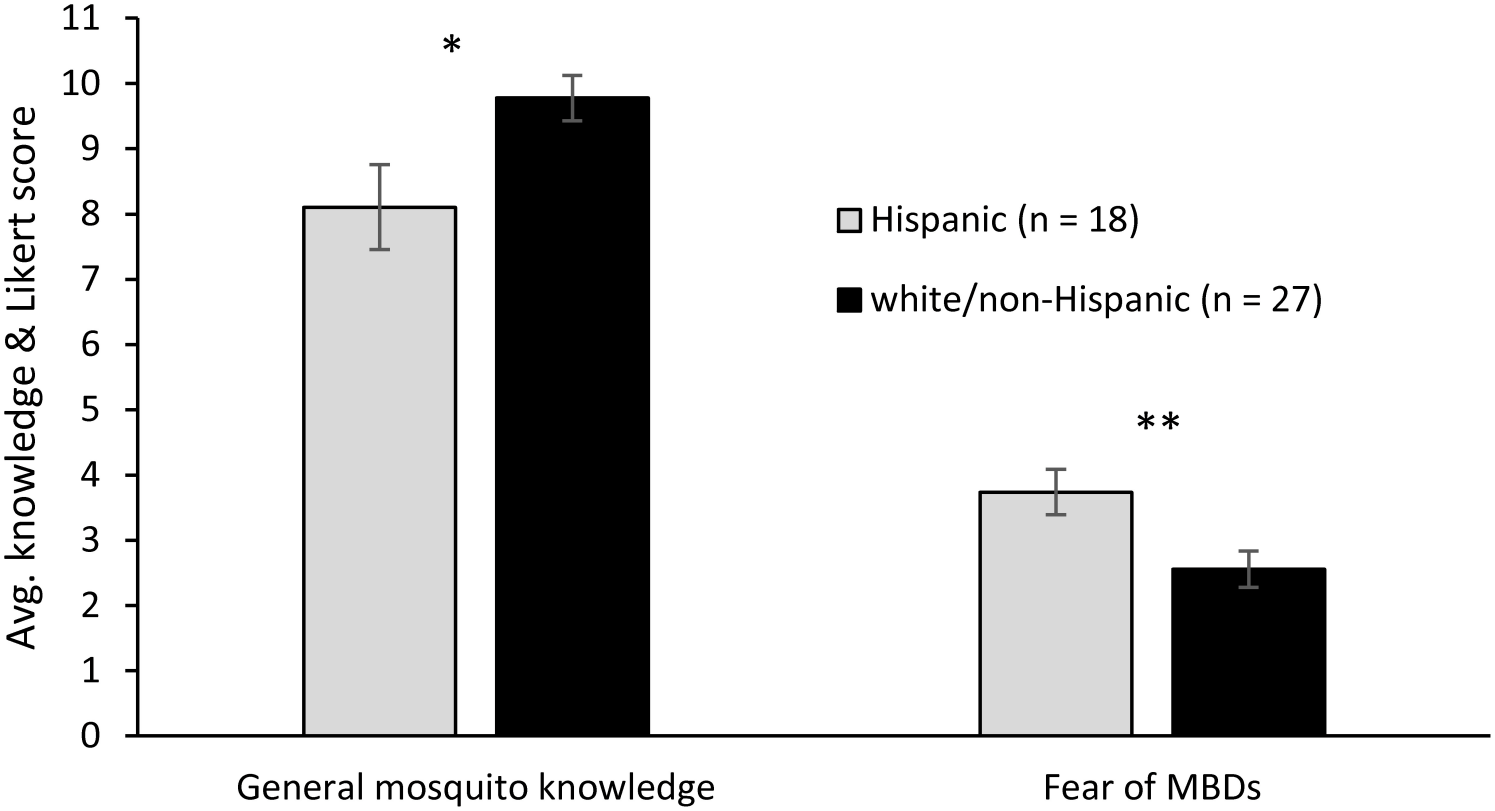
Knowledge and attitudes showing significant differences between Hispanic and white/non-Hispanic respondents. The average knowledge score (y-axis) was calculated by averaging the total number of correct answers on general mosquito knowledge among respondents within each ethnic group. The average Likert score across all statements pertaining to the attitude fear of MBDs was calculated for each respondent, then averaged within ethnic group (y-axis). Individual *t*-tests were performed. *p < 0.05, **p < 0.01.

Knowledge did not correlate with age, income, education, or PCI (Tables 5, 6), but respondents who identified as Hispanic scored significantly lower on general knowledge of mosquitoes than white/non-Hispanic respondents (Figure 2; Supplementary Table 3). When we looked at knowledge and the frequency of PPMs, only one was correlated with any measure of knowledge; the use of insect repellent was positively correlated with WNV knowledge and total sums knowledge (Table 6). Three attitudes, Mosquitoes are a problem, MBDs are serious, and Fear of MBDs positively correlated with some measure of knowledge (Table 5). Both DENV and general mosquito knowledge correlated with MBDs are serious, ZIKV knowledge correlated with the attitude that mosquitoes are a risk and WNV knowledge correlated with fear of MBDs. All other correlations with knowledge were non-significant (Tables 5, 6), including across zones (Supplementary Table 2).

**Table 5:** Correlations between attitudes and personalities, knowledge, practices, and demographics.

	**Attitudes** **Coefficient of correlation (*****P*****-value)**					
	**Mosquitoes are a risk**	**MBDs are serious**	**Fear of Mosquitoes**	**Fear of MBDs**	**The city is doing a good job**	**Yard maintenance is important**
**Personality Trait n** = 49
Extraversion	0.136 (0.352)	−0.027 (0.852)	0.236 (0.102)	0.005 (0.972)	0.095 (0.516)	0.438 (0.002^*^)
Agreeableness	−0.016 (0.910)	0.037 (0.802)	0.278 (0.053)	0.162 (0.267)	0.048 (0.745)	0.092 (0.355)
Conscientiousness	−0.227 (0.116)	−0.006 (0.966)	0.001 (0.993)	−0.113 (0.440)	−0.055 (0.708)	0.528 (0.013^*^)
Neuroticism	0.119 (0.417)	−0.093 (0.523)	0.015 (0.920)	0.238 (0.10)	−0.080 (0.584)	−0.248 (0.086)
Openness	−0.205 (0.157)	0.101 (0.488)	−0.147 (0.315)	−0.253 (0.079)	−0.013 (0.930)	0.041 (0.778)
**Knowledge n** = 50
General mosquito	0.177 (0.219)	0.319 (0.024^*^)	0.240 (0.093)	0.098 (0.496)	0.087 (0.607)	−0.116 (0.422)
Dengue	0.143 (0.322)	0.287 (0.043^*^)	−0.015 (0.916)	0.031 (0.830)	−0.073 (0.613)	−0.213 (0.138)
Zika	0.286 (0.044^*^)	0.264 (0.064)	0.156 (0.281)	0.077 (0.594)	0.036 (0.806)	−0.136 (0.347)
West Nile	0.124 (0.391)	0.148 (0.305)	0.149 (0.303)	0.315 (0.026^*^)	−0.193 (0.179)	0.055 (0.704)
Total sum	0.166 (0.249	0.124 (0.390)	0.076 (0.599)	0.226 (0.115)	−0.143 (0.320)	−0.031 (0.833)
**Practices n** = 45
Wears protective clothes	0.218 (0.136)	0.109 (0.459)	0.048 (0.748)	0.040 (0.788)	0.347 (0.016^*^)	0.163 (0.268)
Uses insect repellent	0.543 (0.000^*^)	0.081 (0.578)	0.099 (0.498)	0.230 (0.112)	−0.130 (0.375)	0.183 (0.208)
Uses insecticides	0.267 (0.069)	0.252 (0.087)	0.321 (0.028^*^)	0.203 (0.171)	−0.155 (0.298)	0.266 (0.070)
Eliminates standing water	−0.098 (0.540)	0.358 (0.021^*^)	−0.131 (0.141)	0.148 (0.357)	0.394 (0.011^*^)	0.103 (0.524)
Uses BTI	0.022 (0.885)	0.193 (0.205)	0.092 (0.549)	0.033 (0.829)	−0.008 (0.960)	0.138 (0.365)
**Demographics**
Age *n* = 44	−0.024 (0.875)	0.324 (0.032^*^)	0.045 (0.773)	0.058 (0.710)	0.493 (0.001^*^)	0.160 (0.298)
Education *n* = 34	0.107 (0.548)	0.045 (0.801)	−0.092 (0.605)	−0.163 (0.357)	−0.025 (0.890)	0.130 (0.561)
Income *n* = 42	−0.161 (0.309)	0.045 (0.777)	−0.160 (0.313)	−0.192 (0.223)	−0.139 (0.380)	0.058 (0.715)
PCI *n* = 47	0.172 (0.247)	0.066 (0.661)	0.128 (0.390)	0.139 (0.350)	−0.101 (0.500)	−0.260 (0.077)

The bolded text with the * indicates the significant values.

* *p* ≥ 0.05.

**Table 6:** Correlations between practices and personalities, knowledge, and demographics.

	**Practices and premise condition index** **Coefficient of correlation** ***(P*****-value)**				
	**Wears protective clothing**	**Uses insect repellant**	**Uses insecticides**	**Eliminates standing water**	**Uses BTI**
**Personality trait n** **=** **49**
Extroversion	0.036 (0.807)	0.045 (0.758)	0.326 (0.026)	−0.069 (0.668)	−0.102 (0.505)
Agreeableness	0.134 (0.366)	−0.224 (0.121)	−0.010 (0.946)	−0.373 (0.016^*^)	−0.141 (0.354)
Conscientiousness	−0.203 (0.167)	−0.049 (0.740)	0.138 (0.356)	0.272 (0.085)	0.099 (0.516)
Neuroticism	0.154 (0.297)	−0.201 (0.166)	−0.250 (0.090)	−0.098 (0.540)	−0.196 (0.196)
Openness	0.094 (0.524)	−0.264 (0.067)	−0.248 (0.093)	−0.305 (0.052)	0.174 (0.252)
**Knowledge n** **=** **50**
General mosquito	0.123 (0.407)	−0.032 (0.828)	0.065 (0.663)	0.169 (0.290)	0.161 (0.291)
Dengue	−0.052 (0.727)	0.157 (0.282)	0.184 (0.214)	0.104 (0.517)	0.131 (0.391)
Zika	0.134 (0.365)	0.149 (0.308)	0.164 (0.271)	0.059 (0.715)	0.165 (0.277)
West Nile	0.086 (0.561)	0.295 (0.039^*^)	0.069 (0.643)	0.028 (0.862)	0.174 (0.253)
Total sum	0.135 (0.359)	0.386 (0.006^*^)	0.081 (0.588)	0.159 (0.714)	0.162 (0.288)
**Demographics**
Age *n* = 44	0.234 (0.131)	0.114 (0.460)	0.149 (0.345)	0.248 (0.145)	−0.036 (0.821)
Education *n* = 34	0.377 (0.028^*^)	0.147 (0.405)	−0.107 (0.552)	−0.008 (0.969)	0.474 (0.007^*^)
Income *n* = 42	−0.134 (0.411)	0.231 (0.140)	0.237 (0.140)	0.028 (0.873)	0.161 (0.334)
PCI *n* = 47	0.031 (0.839)	0.172 (0.254)	−0.055 (0.723)	0.013 (0.937)	0.153 (0.332)

* *P*-value is < 0.05, PCI, Premise Condition Index. The bolded text with the * indicates the significant values.

### Attitudes and personality

**Figure 3:**
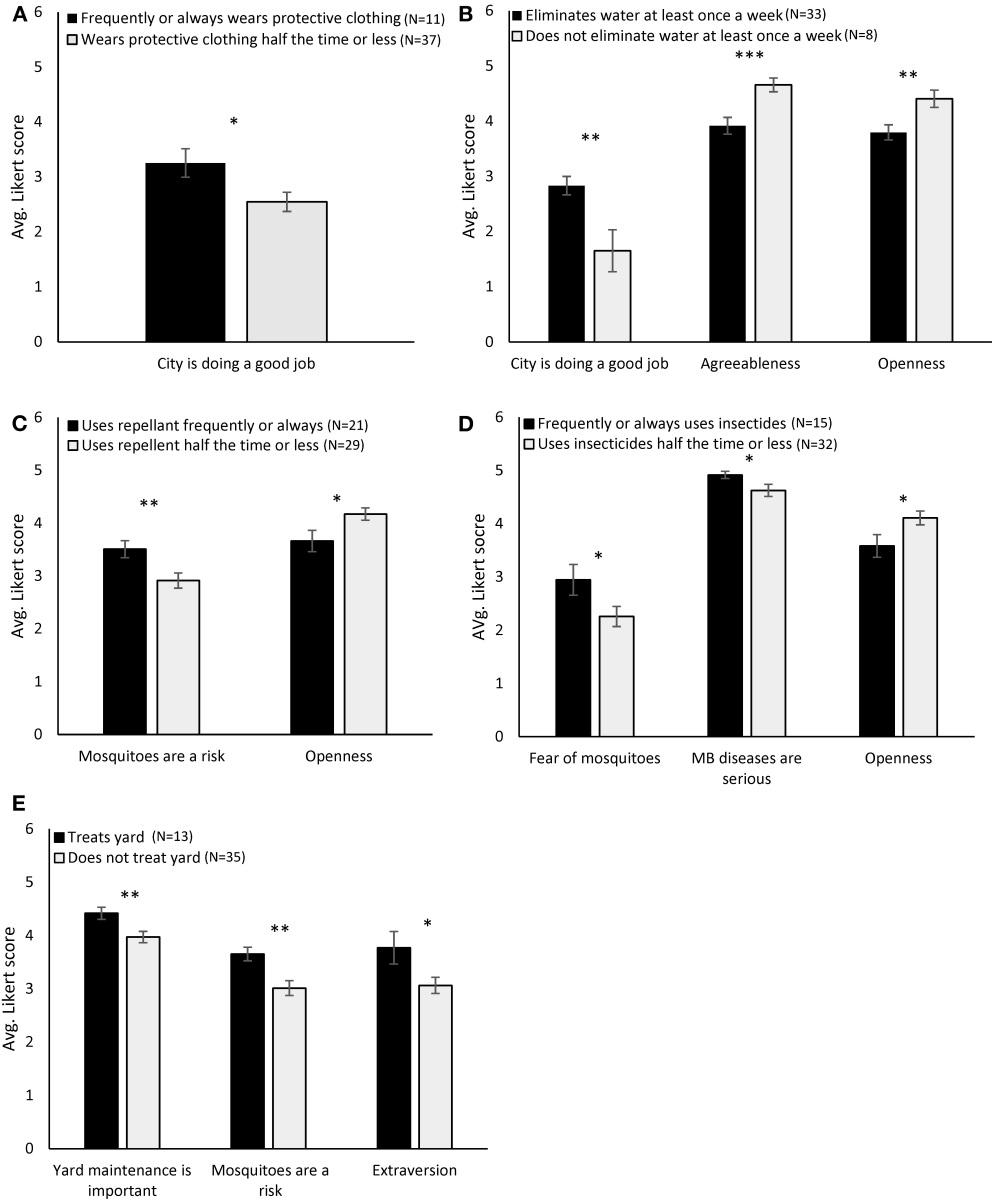
Relationship of dichotomized practices with knowledge, attitudes, and personality. Significant differences were determined by individual *t*-tests where the two groups compared were those that reported appropriate practice of PPMs and those that did not. PPMs were **(A)** Wears protective clothing, **(B)** Eliminates water, **(C)** Uses repellents, **(D)** Uses insecticides, **(E)** Treats yard. *p < 0.05, **p < 0.01, ***p < 0.001.

On average, respondents thought that MBDs were serious and that yard maintenance was important (Table 4). Conversely, respondents, on average did not fear mosquitoes and had no strong feelings regarding fear of MBDs, role of the city, or that mosquitoes are a risk (Table 4). However, 81.6% of respondents considered mosquito activity around their home to be moderate to high. Attitudes did not correlate with income, education, PCI (Table 5), race/ethnicity (Supplementary Table 3), or zones (Supplementary Table 2). The attitude that MBDs are serious positively correlated with five parameters; general mosquito knowledge, dengue knowledge, eliminating standing water, age (Table 5), and appropriate use of insecticides (Figure 3D). The attitude that the city is doing a good job positively correlated with three parameters; wearing protecting clothing, eliminating standing water, and age (Table 5; Figures 3A,B). The attitude that mosquitoes are a risk positively correlated with three parameters; knowledge of Zika, use of insect repellent, and treating the yard (Table 5; Figures 3C,E). Fear of mosquitoes positively correlated with only one parameter; use of insecticides (Table 5; Figure 2D). Likewise, the attitude that yard maintenance was important positively correlated with only one parameter; treating the yard (Figure 3E). However, the attitude that yard maintenance was important was the only attitude that correlated with personality; extraversion and conscientiousness were both positively correlated with this attitude (Table 5).

### Practices and personality

**Figure 4:**
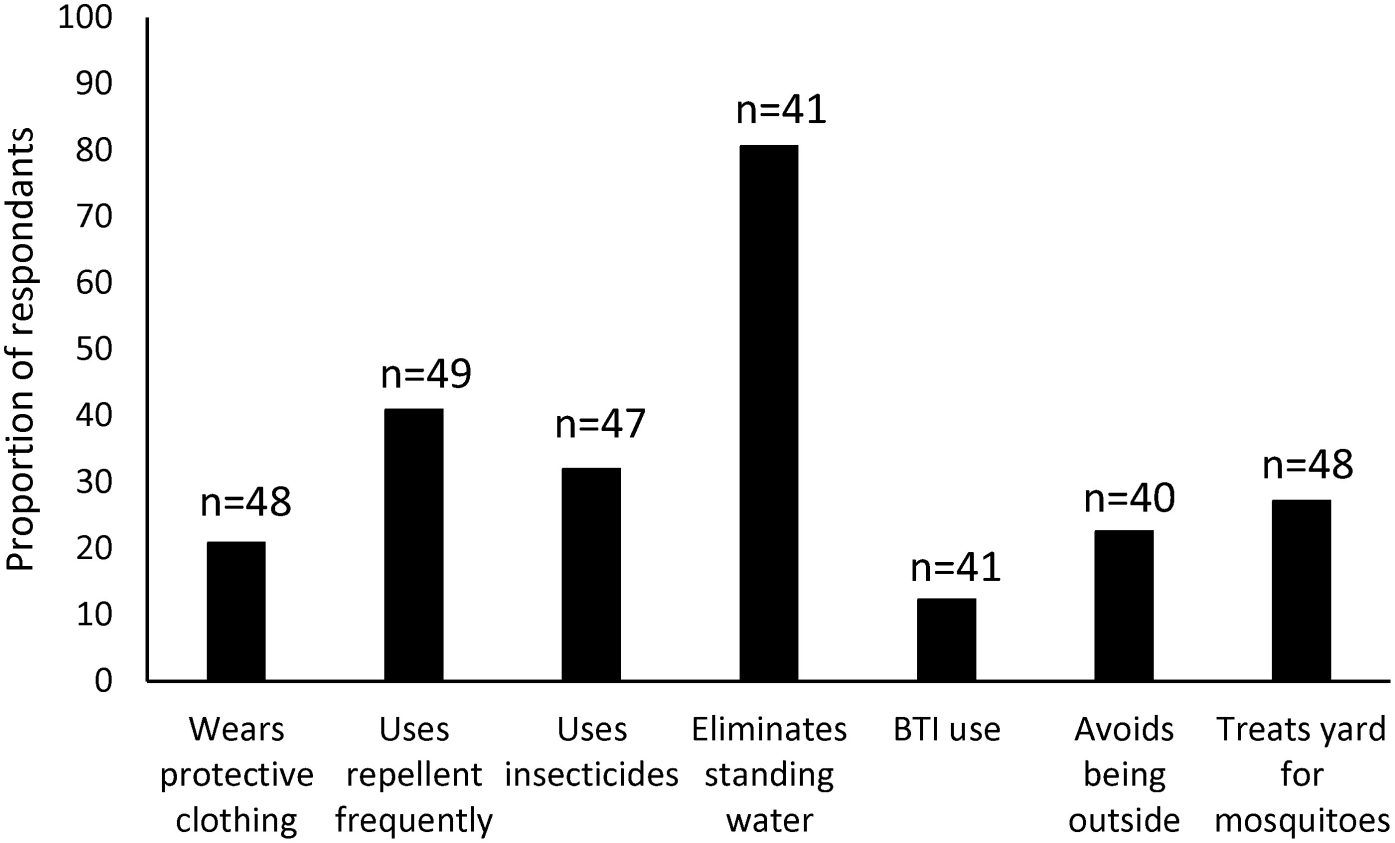
Proportion of respondents self-reporting protective behaviors.

The majority of respondents (80.5%) self-reported correct elimination of standing water and the fewest respondents (12.2%) reported using BTI (Figure 4). Less than 30% of respondents treated their yard for mosquitoes, wore protective clothing, or avoided being outside (Figure 4). When asked why they did not wear protective clothing, 63% responded with some version of “it was too hot.” Neither race/ethnicity nor zone were associated with practicing PPMs (Supplementary Tables 1, 2). The only demographic that correlated with a PPM was education, the higher the education of the participant, the more often they wore protective clothing and used BTI (Table 6).

Three personality traits were associated with practices; agreeableness, openness, and extraversion. Agreeableness and openness were both negatively correlated with PPMs; those who did not empty water at the appropriate frequency scored higher on these two traits than respondents who did (Table 6; Figure 3B). Individuals who did not wear insect repellent nor use insecticides at the appropriate frequency scored higher on openness than those that did these practices appropriately (Figures 3C,D). Extraversion, on the other hand, was positively associated with a PPM, those that treated their yard scored higher on extraversion (Figure 3E). Finally, participants who scored higher on the extraversion scale had lower PCI scores; more extroverted individuals had homes with less suitable habitats for mosquitoes (*r* = −0.315 *p* = 0.033).

We used multiple regression analyses to create models predicting PPMs. Knowledge did not predict any practices regardless of model selection strategy. Two global models using all attitudes or all personalities to predict practices were analyzed. We found that two practices, eliminating water and the use of repellent were predicted by all six attitudes, these were the best fit models for these practices (Table 7; Supplementary Table 4). When we used backward design to remove predictors to evaluate best fit models for the other practices, we found that two additional practices were predicted by attitudes: Insecticide use was predicted by four attitudes (Mosquitoes are a risk, MBDs are serious, city is doing a good job, and yard maintenance is importance) and use of protective clothing was predicted by two attitudes (Mosquitoes are a risk and the City is doing a good job; Table 7). Global models that included all personality traits did not predict any practices (Supplementary Table 5). Using a backward design to find the best fit models we found that two practices were predicted by a combination of fewer personality traits: using insecticides was predicted by openness and extraversion, eliminating water was predicted by openness, agreeableness, and conscientiousness (Table 8).

**Table 7:** Multiple regressions for attitudes predicting practices.

**Practice**	**Predictors (−or + association)**	***df***	***R* ^2^**	***F***	***p***
**Global models**
Eliminates water	Mosquitoes are a risk (+) MBDs are serious (+) City is doing a good job (+)^*^ Yard maintenance is important (+) Fear of MBDs (+) Fear of mosquitoes (−)^*^	6.34	0.343	2.953	0.020
Uses repellent	Mosquitoes are a risk (+)^*^ MBDs are serious (+) City is doing a good job (−) Yard maintenance is important (+) Fear of MBDs (+) Fear of mosquitoes (−)^*^	6.42	0.384	4.368	0.002
**Other models**
Use of insecticides	Mosquitoes are a risk (+) MBDs are serious (+) City is doing a good job (−) Yard maintenance is important (+)	4.42	0.219	2.943	0.031
Wears protective clothing	Mosquitoes are a risk (+)^*^ City is doing a good job (+)^*^	2.45	0.207	5.876	0.005

* p < 0.05.

**Table 8:** Multiple regressions for personalities predicting practices.

**Practice**	**Predictors (−or + association)**	***df***	**R^2^**	***F***	***p***
**Other models**
Use of insecticides	Openness (−)^*^ Extraversion (+)^*^	2.44	0.189	5.137	0.010
Eliminates water	Openness (−) Agreeableness (−) Conscientiousness (+)	3.37	0.237	3.824	0.018

* p < 0.05.

## Discussion

Educating the public about mosquitoes and MBDs has been a primary goal of public health entities with the idea that the more one knows, the more likely they are to comply with preventative measures (PPMs). However, in the U.S. results have been mixed as to whether this strategy works. Some studies have shown that knowledge and awareness improve compliance (7, 47) while others show a negative effect (4) or no effect (49). Our study supports the latter; knowledge of mosquitoes and MBDs did not influence the practice of most PPMs. It may be because the overwhelming majority of San Antonio respondents had considerable knowledge about all three MBDs, a similar finding in other communities in the U.S. (5, 9, 10, 49). The majority of our respondents trusted SAMHD for information about mosquitoes and 80% of respondents reported that they emptied their water at least once a week, possibly because SAMHD instituted a media campaign to educate the community about mosquitoes and the risk of contracting ZIKV. This finding also indicates respondents likely knew eliminating water was important in preventing MBDs and thus reported that they did this behavior (28), however this is only a self-reported behavior and Averett et al. (53) found that more people reported knowing they should do a behavior than actually doing it. In the future, we would like to incorporate a rating system of PPMs to assess their perceived importance and to implement larval mosquito surveys to evaluate whether self-reported behavior translates to performing it (4, 7, 10, 49). Also, 90% of San Antonio respondents agreed that MBDs were serious. This is much higher than respondents in the Lower Rio Grande Valley of Texas, where, despite a relatively high level of knowledge about ZIKV and known local transmission, only 35% thought it was serious (10).

We found conditions under which attitudes predicted self-reported practices. All practices were predicted by or correlated with at least one attitude measure of perceived risk (MBDs are serious, Mosquitoes are a risk) or fear (fear of MBDs, fear of mosquitoes). Overall, there was a positive association with perceived risk, fear, and practicing PPMs (Figure 3; Table 6). The interpretation of these results is intuitive; it stands to reason that the more one fears or perceives risk, the more likely they are to employ practices that reduce or eliminate the fear and risk. For example, Herrington (54) found that concern over being bitten was the strongest predictor of appropriate PPMs and Harper et al. (55) found that fear was the only predictor of positive behavior change as it related to COVID-19 prevention. Likewise, perceived risk impacts compliance with mosquito PPMs; in addition, perceived risk differs with geographic location and previous history of local transmission of MBDs (2, 7, 56). While we saw a positive correlation of fear and perceived risk with PPMs, our respondents, on average, did not fear mosquitoes or MBDs, in fact, the majority strongly disagreed with statements about those factors. Also, fear negatively predicted emptying water; it may be that those emptying water might not fear MBDs because they know they are already conducting appropriate PPMs. Thus, there are nuances in how fear and perceived risk might motivate behavioral compliance in San Antonio; this highlights the need to continue to include both factors in KAP studies in order to develop appropriate community-specific messaging.

Another attitude that predicted or correlated with multiple practices was whether or not participants thought that the city was doing a good job in mosquito control and education. We suggest that compliance with PPMs by people who think the city is doing a good job is consistent with the idea that people are conditional cooperators; they are more likely to cooperate when they think others are also cooperating. Women in Kenya and Ghana were more willing to use bed nets if they knew neighbors had them or were also using them and that doing so would provide both a private and public benefit to their community (57, 58). In our study, the perception that the city is doing their part in mosquito control and education would be similar to the perception that others in the community were participating and thus, could influence whether community members participate in PPMs. Additionally, if residents consider themselves group members in the city, and they have positive feelings toward the city's handling of mosquito issues, they may be more likely themselves to cooperate. This is similar to social identity theory as it relates to teams where positive feelings toward a team results in team identification and thus cooperation (59) or where positive feelings within a group fosters more teamwork and encourages members to do things for the group (60). Therefore, people join the city's “team” and cooperate when they think the city is doing well, and participate in self-serving practices when they believe the opposite.

Personality factors can help predict health-related behaviors (16) by providing insight into identifying the factors that determine perceptions of risk and fear as they pertain to the target of the behaviors (19). In our study we found that openness, agreeableness, and extraversion were correlated with several PPMs. Both openness and agreeableness negatively correlated with PPMs; those who scored high on openness and agreeableness did not empty water appropriately and those who scored high on openness did not use insecticides frequently. We did not expect to find these negative correlations as these two traits are consistently associated with practicing healthy behaviors and avoiding risky ones (16, 61–63) with a few exceptions (31, 61, 64). For example, Abdelrahman (64) found that agreeableness was negatively associated with social distancing. He suggested that because altruism, empathy, and helping behavior are characteristics of agreeableness, individuals may prioritize social interactions above social distancing. Similarly, extraversion is usually correlated with risky behaviors such as substance abuse, unsafe driving, and risky sex practices, but it is also positively correlated with “general” healthy behavior (16). In our study we found that those who scored high on extraversion treated their yard frequently. Perhaps the need to be social at one's home motivated the extraverted individual to ensure their yard was safe for guests. This interpretation is supported by our finding that extraversion and the attitude that yard maintenance is important were positively correlated. These examples highlight the need to understand the behaviors themselves, the ramifications of that behavior, and the interpretation of the personality correlated with that behavior.

The addition of personality traits to a KAP study must be useful and we should ask how might it assist in the messaging used by public health officials to increase compliance with behaviors The COVID-19 pandemic provides numerous opportunities to evaluate messaging and behavioral compliance (32, 64–66). Krupić et al. (32) used both a personality survey and a questionnaire of Approach and Avoidance Motivation to understand how personality, messaging, and behavioral compliance may be linked during a community-wide campaign to reduce the spread of COVID-19. They found that agreeable and conscientious people were most compliant and responded best to approach-motivation (positive outcomes as a result of complying) rather than avoidance-motivation (highlighting dangerousness if not compliant). Tailoring messages aimed at persuasion to comply are not new (67, 68) and studies such as these make the case for the usefulness of incorporating personality surveys into KAP studies.

### Limitations

The most significant limitation of this study was the low response rate (10.6%) and the resulting small sample size, 50 participants, used to represent KAP of residents in such a highly populous city. However, a response rate this low is not atypical, Samuel et al. (6) reported a 15% response rate in Alabama (126 respondents) and Mitchell et al. (69) reported a 12.4% response rate in Maryland. Richards et al. (5) offered a $5 gift card upon completion of a 27-question survey in North Carolina and their response rate was 34%. It is possible that offering a monetary incentive may increase response rate in future studies in San Antonio, especially in areas that are presumably low SES. Juarez et al. (10) had a maximum of 39 respondents in their KAP analysis along the border of Texas, likely due to the effort in pairing the KAP survey with intensive mosquito collections. Another limitation is that the respondents were mostly high-income and older. Additionally, our door-to-door surveys were conducted during the hottest time of the year in San Antonio and for the safety of our research assistants, all surveys were completed outdoors. While we carried out the surveys during the morning hours, the length of our survey (96 questions), which took about 30 min to complete, may have limited the willingness of residents to stand outdoors to complete these surveys.

## Conclusion

This is the first study to evaluate the implications of different personality types on behaviors associated with preventing mosquitoes and MBDs. We found that three personality traits were associated with practicing mosquito-related PPMs and that they were useful in predicting the performance of some PPMs. Because studies have shown that personality measures are relevant to effective public health messaging and because we found initial evidence that personality influences compliance with mosquito-related PPMs, we suggest that incorporating personality measures into a KAP study is an easy addition that has the potential to improve our ability to tailor public health messaging in ways that increase compliance. We also found that self-reported PPMs did not differ along ethnic or neighborhood lines and that attitudes toward the role of the city was an important factor in predicting PPMs, suggesting that in San Antonio, city culture (attitudes common throughout the city as opposed to attitudes differing by ethnicity and neighborhood) may be most salient in developing public health messaging. In sum, our study demonstrates a needed contribution to the literature in mosquito prevention due to the geographic location (a highly populous ecologically diverse and economically segregated urban setting with a Hispanic majority), the addition of a robust attitude measure, and the inclusion of personality variables known to predict health behaviors.

## Data availability statement

The raw data supporting the conclusions of this article will be made available by the authors, without undue reservation.

## Ethics statement

Human subject approval was provided by A&M-SA Institutional Review Board (protocol # 2018-49). Written informed consent for participation was not required for this study in accordance with the national legislation and the institutional requirements.

## Author contributions

AB: reviewed the personality and attitude portions of the KAPP survey, analyzed survey data, co-wrote manuscript, and project managed survey. LM-B and AS equally participated in the following: wrote draft of the KAPP survey, conducted survey, entered survey data, and conducted literature search. MW: reviewed the knowledge, attitudes, practice portions of the KAPP survey, analyzed survey data, constructed figures and tables, co-wrote manuscript, and project managed survey. All authors contributed to the article and approved the submitted version.

## Funding

Funding was provided by an A&M-SA undergraduate research grant: FIRE-UP (Fostering Interdisciplinary Research to improve Undergraduate Performance) awarded to LM-B and AS.

## Conflict of interest

The authors declare that the research was conducted in the absence of any commercial or financial relationships that could be construed as a potential conflict of interest.

## Publisher's note

All claims expressed in this article are solely those of the authors and do not necessarily represent those of their affiliated organizations, or those of the publisher, the editors and the reviewers. Any product that may be evaluated in this article, or claim that may be made by its manufacturer, is not guaranteed or endorsed by the publisher.

## Supplementary material

Supplementary Figure 1 The process of how likert-scored questions were dichotomized for whether respondents practiced a behavior appropriate for preventing mosquitoes and MBDs.

## References

[1] World Health Organization. Knowledge attitudes and practice surveys: Zika virus disease and potential complications: resource pack. (2016). Available online at: https://apps.who.int/iris/handle/10665/204689 (accessed December 13, 2021).

[2] Zielinski-Gutierrez EC Hayden MH. A model for defining West Nile virus risk perception based on ecology and proximity. Ecohealth. (2006) 3:28–34. 10.1007/s10393-005-0001-9

[3] Brown JA Larson KL Lerman SB Cocroft A Hall SJ. Resident perceptions of mosquito problems are more influenced by landscape factors than mosquito abundance. Sustainability. (2021) 13:2011533. 10.3390/su132011533

[4] Bodner D LaDeau SL Biehler D Kirchoff N Leisnham PT. Effectiveness of print education at reducing urban mosquito infestation through improved resident-based management. PLoS ONE. (2016) 11:e0155011. 10.1371/journal.pone.0155011 27171195 PMC4865130

[5] Richards SL Balanay JA Byrd BD Reiskind MH Styers DM. Regional survey of mosquito control knowledge and usage in North Carolina. J Am Mosq Control Assoc. (2017) 33:331–9. 10.2987/17-6669.1 29369034

[6] Samuel G DiBartolo-Cordovano R Taj I Merriam A Lopez JM Torres C. A survey of the knowledge, attitudes and practices on Zika virus in New York City. BMC Public Health. (2018) 18:1–11. 10.1186/s12889-017-4991-3 29291723 PMC5748954

[7] Haenchen SD Hayden MH Dickinson KL Walker K Jacobs EE Brown HE. Mosquito avoidance practices and knowledge of arboviral diseases in cities with differing recent history of disease. Am J Trop Med Hyg. (2016) 95:945–53. 10.4269/ajtmh.15-0732 27527634 PMC5062805

[8] Walker KR Williamson D Carrière Y Reyes-Castro PA Haenchen S Hayden MH. Socioeconomic and human behavioral factors associated with *Aedes aegypti* (Diptera: Culicidae) immature habitat in Tucson, AZ. J Med Entomol. (2018) 55:955–63. 10.1093/jme/tjy011 29471405 PMC6025186

[9] Morse W Izenour K McKenzie B Lessard S Zohdy S. Perceptions and practices of mosquito-borne diseases in Alabama–is concern where it should be? BMC public health. (2019) 19:1–9. 10.1186/s12889-019-7308-x 31337359 PMC6652104

[10] Juarez JG Garcia-Luna S Medeiros MC Dickinson KL Borucki MK Frank M. The eco-bio-social factors that modulate *Aedes aegypti* abundance in South Texas Border Communities. Insects. (2021) 12:183. 10.3390/insects12020183 33670064 PMC7926310

[11] Centers for Disease Control ArboNET. Disease maps 2010–2021 Chikungunya, Dengue, Zika, and West Nile human cases (2021). Available online at: https://wwwncdcgov/arbonet/maps/ADB_Diseases_Map/indexhtml (accessed December 13, 2021).

[12] Reiter P. Climate change and mosquito-borne disease. Environ Health Perspect. (2001) 109:141–61. 10.1289/ehp.01109s1141 11250812 PMC1240549

[13] Hatcher L. Advanced Statistics in Research. Saginaw, MI: Shadow Finch Media (2013).

[14] Faulkenberry TJ. Psychological Statistics: The Basics. New York: Routledge (2022).

[15] McCrae RR Costa PT Jr. The five-factor theory of personality. In:John OP Robins RW and Pervin LA, Editors. Handbook of personality: Theory and research. 3^rd^ ed. New York, NY: The Guilford Press (2008). p. 159–81.

[16] Bermúdez J. Personality and health-protective behaviour. Eur J Pers. (1999) 13:83–103.

[17] Conner M Norman P. Predicting and changing health behaviour: Future directions. In:Conner M Norman P, editors. Predicting Health Behaviour. 2nd ed. Berkshire: Open University Press; McGraw-Hll Education (2005). p. 324–71.

[18] McCrae RR Costa PT. Cross-cultural perspectives on adult personality trait development. In:Mroczek DK Little TD, editors. Handbook of Personality Development. Mahwah, NJ: Lawrence Erlbaum Associates (2006). p. 129–45.

[19] Skøt L Nielsen JB Leppin A. Who perceives a higher personal risk of developing type 2 diabetes? a cross-sectional study on associations between personality traits, health-related behaviours and perceptions of susceptibility among university students in Denmark. BMC Public Health. (2018) 18:1–10. 10.1186/s12889-018-5884-9 30075710 PMC6076414

[20] Hall PA Fong GT Epp LJ. Cognitive and personality factors in the prediction of health behaviors: an examination of total, direct and indirect effects. J Behav Med. (2014) 37:1057–68. 10.1007/s10865-013-9535-4 24072429

[21] Lemos-Giráldez S Fidalgo-Aliste AM. Personality dispositions and health-related habits and attitudes: a cross-sectional study. Eur J Pers. (1997) 11:197–209.18028584

[22] Hong RY Paunonen SV. Personality traits and health-risk behaviours in university students. European Journal of Personality: Published for the European Association of Personality Psychology. (2009) 23:675–96. 10.1002/per.736

[23] Raynor DA Levine H. Associations between the five-factor model of personality and health behaviors among college students. J Am Coll Health. (2009) 58:73–82. 10.3200/JACH.58.1.73-82 19592356

[24] Bogg T Milad E. Demographic, personality, and social cognition correlates of coronavirus guideline adherence in a US sample. Health Psychology. (2020) 39:1026–36. 10.1037/hea0000891 33252928

[25] Rochefort C Hoerger M Turiano NA Duberstein P. Big five personality and health in adults with and without cancer. J Health Psychol. (2019) 24:1494–504. 10.1177/1359105317753714 29355050 PMC7427406

[26] Qian K Yahara T. Mentality and behavior in COVID-19 emergency status in Japan: influence of personality, morality and ideology. PLoS ONE. (2020) 15:e0235883. 10.1371/journal.pone.0235883 32649687 PMC7351180

[27] Otterbring T Festila A. Pandemic prevention and personality psychology: gender differences in preventive health behaviors during COVID-19 and the roles of agreeableness and conscientiousness. Journal of Safety Science and Resilience. (2022) 3:87–91. 10.1016/j.jnlssr.2021.11.003 PMC8639385 40477760

[28] Nudelman G Ivanova E. The relationship between frequency of performance and perceived importance of health behaviours. J Health Psychol. (2020) 25:1692–706. 10.1177/1359105318770724 29692209

[29] Armon G Toker S. The role of personality in predicting repeat participation in periodic health screening. J Pers. (2013) 81:452–64. 10.1111/jopy.12021 23126563

[30] Carvalho LD Pianowski G Gonçalves AP. Personality differences and COVID-19: are extroversion and conscientiousness personality traits associated with engagement with containment measures? Trends Psychiatry Psychother. (2020) 42:179–84. 10.1590/2237-6089-2020-0029 32294713

[31] Bahat E. The big five personality traits and adherence to breast cancer early detection and prevention. Pers Individ Dif. (2021) 172:e110574. 10.1016/j.paid.2020.110574

[32] Krupić D Žuro B Krupić D. Big five traits, approach-avoidance motivation, concerns and adherence with COVID-19 prevention guidelines during the peak of pandemic in Croatia. Pers Individ Dif. (2021) 179:110913. 10.1016/j.paid.2021.110913 33850340 PMC8031466

[33] Sheeran P Maki A Montanaro E Avishai-Yitshak A Bryan A Klein WM. The impact of changing attitudes, norms, and self-efficacy on health-related intentions and behavior: a meta-analysis. Health psychology. (2016) 35:1178–88. 10.1037/hea0000387 27280365

[34] Rosenberg MJ. A structural theory of attitude dynamics. Public Opin Q. (1960) 24:319–40. 10.1086/266951

[35] Eagly AH Chaiken S. The Psychology of Attitudes. Orlando, FL: Harcourt Brace Jovanovich College Publishers. (1993) p. 794.

[36] Marsh HW Hau KT Balla JR Grayson D. Is more ever too much? The number of indicators per factor in confirmatory factor analysis. Multivariate Behav Res. (1998) 33:181–220. 10.1207/s15327906mbr3302_1 26771883

[37] Robinson MA. Using multi-item psychometric scales for research and practice in human resource management. Hum Resour Manage. (2018) 57:739–50. 10.1002/hrm.21852

[38] Gliem JA Gliem RR. Calculating, interpreting, and reporting Cronbach's alpha reliability coefficient for Likert-type scales. Columbus, OH: Midwest Research-to-Practice Conference in Adult, Continuing, and Community Education (2003).

[39] Rummel RJ. Applied Factor Analysis. Evanston, IL: Northwestern University Press (1970).

[40] Tun-Lin W Kay BH Barnes AN. The premise condition index: a tool for streamlining surveys of *Aedes aegypti*. Am J Trop Med Hyg. (1995) 53:591–4. 10.4269/ajtmh.1995.53.591 8561259

[41] Bland JM Altman DG. Statistics notes: Cronbach's alpha. BMJ. (1997) 314:572. 10.1136/bmj.314.7080.572 9055718 PMC2126061

[42] United States Census Bureau. 2020 Decennial Census (2021). Available online at: https://data.census.gov/cedsci/ (accessed December 13, 2021).

[43] Florida R Mellander C. Segregated City: The Geography of Economic Segregation in America's Metros. Toronto, ON: Martin Prosperity Institute (2015). p. 86.

[44] Griffith GE Bryce SA Omernik JM Rogers A. Ecoregions of Texas. Austin: Texas Commission on Environmental Quality. Report (2007). p. 125

[45] Wise de Valdez MR. Mosquito species distribution across urban, suburban, and semi-rural residences in San Antonio, Texas. J Vector Ecol. (2017) 42:184–8. 10.1111/jvec.12254 28504436

[46] Obregón JA Ximenez MA Villalobos EE de Valdez MRW. Vector mosquito surveillance using centers for disease control and prevention autocidal gravid ovitraps in San Antonio, Texas. J Am Mosq Control Assoc. (2019) 35:178–85. 10.2987/18-6809.1 31647715

[47] Dowling Z Armbruster P LaDeau SL DeCotiis M Mottley J Leisnham PT. Linking mosquito infestation to resident socioeconomic status, knowledge, and source reduction practices in suburban Washington, DC. Ecohealth. (2013)10:36–47. 10.1007/s10393-013-0818-6 23377982

[48] Wise de Valdez MR Phillips J Obregón JA Aloba A. Adult mosquito distribution associated with premise condition index and socioeconomics of neighborhoods in San Antonio, TX. In Preparation.

[49] Tuiten W Koenraadt CJ McComas K Harrington LC. The effect of West Nile virus perceptions and knowledge on protective behavior and mosquito breeding in residential yards in upstate New York. Ecohealth. (2009) 6:42–51. 10.1007/s10393-009-0219-z 19452223

[50] Donnellan MB Oswald FL Baird BM Lucas RE. The mini-IPIP scales: tiny-yet-effective measures of the big five factors of personality. Psychol Assess. (2006) 18:192–203. 10.1037/1040-3590.18.2.192 16768595

[51] Vittinghoff E McCulloch CE. Relaxing the rule of ten events per variable in logistic and cox regression. Am J Epidemiol. (2007) 165:710–8. 10.1093/aje/kwk052 17182981

[52] Austin PC Steyerberg EW. The number of subjects per variable required in linear regression analyses. J Clin Epidemiol. (2015) 68:627–36. 10.1016/j.jclinepi.2014.12.014 25704724

[53] Averett E Neuberger JS Hansen G Fox MH. Evaluation of West Nile virus education campaign. Emerg Infect Dis. (2005) 11:1751–3. 10.3201/eid1111.050363 16318730 PMC3367363

[54] Herrington JE Jr. Pre-West Nile virus outbreak: perceptions and practices to prevent mosquito bites and viral encephalitis in the United States. Vector-borne and zoonotic diseases. (2003) 3:157–73. 10.1089/153036603322662156 14733669

[55] Harper CA Satchell LP Fido D Latzman RD. Functional fear predicts public health compliance in the COVID-19 pandemic. Int J Ment Health Addict. (2021) 19:1875–88. 10.1007/s11469-020-00281-5 32346359 PMC7185265

[56] Castro M Sánchez L Pérez D Sebrango C Shkedy Z Van der Stuyft P. The relationship between economic status, knowledge on dengue, risk perceptions and practices. PLoS ONE. (2013) 8:e81875. 10.1371/journal.pone.0081875 24349145 PMC3861357

[57] Ernst KC Erly S Adusei C Bell ML Kessie DK Biritwum-Nyarko A. Reported bed net ownership and use in social contacts is associated with uptake of bed nets for malaria prevention in pregnant women in Ghana. Malar J. (2017) 16:13. 10.1186/s12936-016-1660-4 28049471 PMC5210303

[58] Gatua JG. Information and cooperation in preventive health behavior: the case of bed net use in rural Kenya. Health Econ. (2021) 30:2124–43. 10.1002/hec.4365 34096642

[59] Lin CP He H Baruch Y Ashforth BE. The effect of team affective tone on team performance: the roles of team identification and team cooperation. Hum Resour Manage. (2017) 56:931–52. 10.1002/hrm.21810

[60] Peñalver J Salanova M Martínez IM Schaufeli WB. Happy-productive groups: how positive affect links to performance through social resources. J Posit Psychol. (2019) 14:377–92. 10.1080/17439760.2017.1402076

[61] Bessey D. Preferences, personality and health behaviors: results from an explorative economic experiment. Int J Health Econ Manag. (2018) 18:437–56. 10.1007/s10754-018-9236-1 29476285

[62] Jokela M Airaksinen J Kivimäki M Hakulinen C. Is within–individual variation in personality traits associated with changes in health behaviours? Analysis of seven longitudinal cohort studies. Eur J Pers. (2018) 32:642–52. 10.1002/per.2173

[63] Han H. Exploring the association between compliance with measures to prevent the spread of COVID-19 and big five traits with Bayesian generalized linear model. Pers Individ Differ. (2021) 176:110787. 10.1016/j.paid.2021.110787 33642661 PMC7901385

[64] Abdelrahman M. Personality traits, risk perception, and protective behaviors of Arab residents of Qatar during the COVID-19 pandemic. Int J Ment Health Addict. (2020) 22:1–2. 10.31234/osf.io/6g7kh 32837433 PMC7307935

[65] Blagov PS. Adaptive and dark personality in the COVID-19 pandemic: predicting health-behavior endorsement and the appeal of public-health messages. Soc Psychol Personal Sci. (2021) 12:697–707. 10.1177/1948550620936439 PMC7342937 38602980

[66] Heffner J Vives ML FeldmanHall O. Emotional responses to prosocial messages increase willingness to self-isolate during the COVID-19 pandemic. Pers Individ Dif. (2021) 170:110420. 10.1016/j.paid.2020.110420 33082614 PMC7561320

[67] Halko S Kientz JA. Personality and persuasive technology: an exploratory study on health-promoting mobile applications. In: Persuasive technology. Berlin: Springer Berlin Heidelberg (2010). p 150–61. 10.1007/978-3-642-13226-1_16

[68] Hirsh JB Kang SK Bodenhausen GV. Personalized persuasion: tailoring persuasive appeals to recipients' personality traits. Psychol Sci. (2012) 23:578–81. 10.1177/0956797611436349 22547658

[69] Mitchell KC Ryan P Howard DE Feldman KA. Understanding knowledge, attitudes, and behaviors toward West Nile Virus prevention: a survey of high-risk adults in Maryland. Vector-Borne and Zoonotic Diseases. (2018) 18:173–80. 10.1089/vbz.2017.2188 29336697

