# Supplementary figures and images for: Personality and city culture predict attitudes and practices toward mosquitoes and mosquito-borne diseases in South Texas
Source: Front Public Health. 2022 Nov 7;10:919780. doi: 10.3389/fpubh.2022.919780 (PMC9676665; doi:10.3389/fpubh.2022.919780)

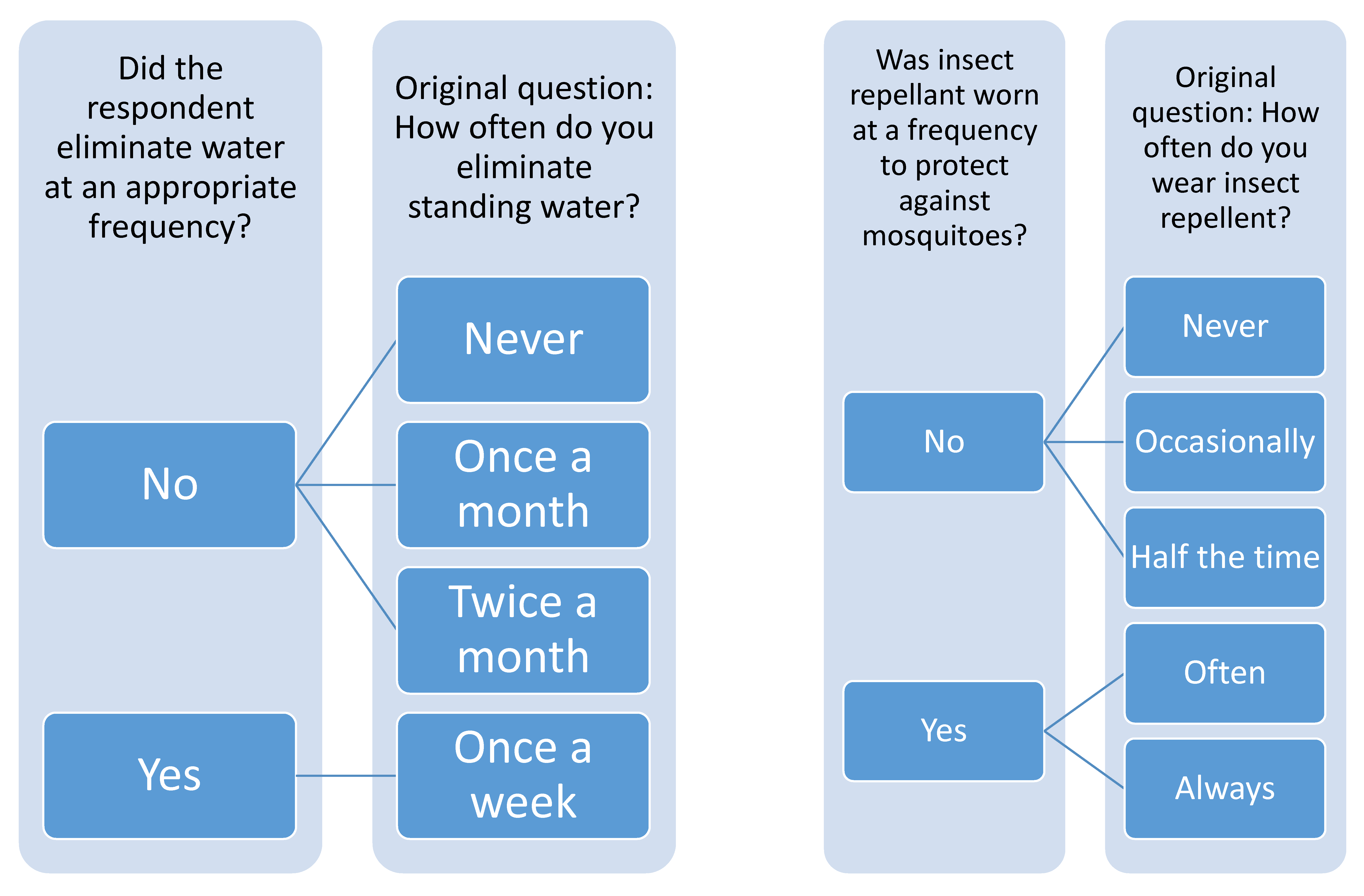

Supplement: Supplementary Figure 1 — The process of how likert-scored questions were dichotomized for whether respondents practiced a behavior appropriate for preventing mosquitoes and MBDs. [file Image_1.tif]
