# Supplementary material for: Personality and city culture predict attitudes and practices toward mosquitoes and mosquito-borne diseases in South Texas
Source: Front Public Health. 2022 Nov 7;10:919780. doi: 10.3389/fpubh.2022.919780 (PMC9676665; doi:10.3389/fpubh.2022.919780)
Supplement: Supplementary file 2 [file Data_Sheet_1.docx]

| Supplemental Table 1. Chi-squared and *t*-tests with non-significant results of race/ethnicity with practices, demographics. | | |
| --- | --- | --- |
| KAP parameter | *X*^2^ (df) | *p*-value |
| Practices (dichotomized): X^2^ |  |  |
| Eliminates water | 0.938 (1) | 0.333 |
| Uses repellent | 0.037 (1) | 0.848 |
| Uses insecticides | 1.346 (1) | 0.246 |
| Wears protective clothing | 0.023 (1) | 0.880 |
| Treats yard | 0.009 (1) | 0.923 |
| Education | 7.408 (5) | 0.192 |
| Income | 11.132 (8) | 0.194 |
| Zone | 0.550 (2) | 0.760 |
| Practices (frequency scale): *t*-test | df | *p*-value |
| Eliminates water | 35 | 0.537 |
| Uses repellent | 43 | 0.923 |
| Uses insecticides | 41 | 0.478 |
| Wears protective clothing | 42 | 0.611 |

| Supplemental Table 2. ANOVA comparing the three San Antonio areas where KAPP survey was conducted. | | | | | | |
| --- | --- | --- | --- | --- | --- | --- |
| KAP Parameters |  | Sum of Square | df | Mean Square | F | *p*-value |
| ***Demographics*** |  |  |  |  |  |  |
| Age | Between Groups | 100.8 | 2 | 50.42 | 0.17 | 0.85 |
|  | Within Groups | 11627.07 | 39 | 298.13 |  |  |
|  | Total | 11727.91 | 41 |  |  |  |
| Income | Between Groups | 26.14 | 2 | 13.07 | 2.76 | 0.08 |
|  | Within Groups | 180.35 | 38 | 4.75 |  |  |
|  | Total | 206.49 | 40 |  |  |  |
| Education | Between Groups | 0.28 | 2 | 0.14 | 0.04 | 0.96 |
|  | Within Groups | 107.60 | 29 | 3.71 |  |  |
|  | Total | 107.88 | 31 |  |  |  |
| PCI | Between Groups | 2.165 | 2 | 1.083 | .363 | .698 |
|  | Within Groups | 131.32 | 44 | 2.99 |  |  |
|  | Total | 133.49 | 46 |  |  |  |
| ***Attitudes*** |  |  |  |  |  |  |
| Mosquitoes are a risk | Between Groups | 0.34 | 2 | 0.17 | 0.24 | 0.78 |
|  | Within Groups | 30.22 | 44 | 0.69 |  |  |
|  | Total | 30.56 | 46 |  |  |  |
| MBDs are serious | Between Groups | 0.37 | 2 | 0.19 | 0.83 | 0.44 |
|  | Within Groups | 9.86 | 44 | 0.22 |  |  |
|  | Total | 10.23 | 46 |  |  |  |
| Fear of MBDs | Between Groups | 2.17 | 2 | 1.09 | 0.45 | 0.64 |
|  | Within Groups | 107.46 | 44 | 2.44 |  |  |
|  | Total | 109.63 | 46 |  |  |  |
| Fear of mosquitoes | Between Groups | 0.81 | 2 | 0.41 | 0.33 | 0.72 |
|  | Within Groups | 54.56 | 44 | 1.24 |  |  |
|  | Total | 55.37 | 46 |  |  |  |
| The city is doing a good job | Between Groups | 1.24 | 2 | 0.62 | 0.52 | 0.60 |
|  | Within Groups | 52.67 | 44 | 1.20 |  |  |
|  | Total | 53.91 | 46 |  |  |  |
| Yard maintenance is important | Between Groups | 1.72 | 2 | 0.86 | 3.10 | 0.06 |
|  | Within Groups | 12.21 | 44 | 0.28 |  |  |
|  | Total | 13.94 | 46 |  |  |  |
| ***Knowledge*** |  |  |  |  |  |  |
| Gen. mosquito knowledge | Between Groups | 4.99 | 2 | 2.50 | 0.45 | 0.64 |
|  | Within Groups | 246.96 | 44 | 5.61 |  |  |
|  | Total | 251.96 | 46 |  |  |  |
| DENV knowledge | Between Groups | 56.84 | 2 | 28.42 | 1.38 | 0.26 |
|  | Within Groups | 909.80 | 44 | 20.68 |  |  |
|  | Total | 966.64 | 46 |  |  |  |
| ZIKV Knowledge | Between Groups | 5.32 | 2 | 2.66 | .15 | 0.86 |
|  | Within Groups | 786.30 | 44 | 17.87 |  |  |
|  | Total | 791.62 | 46 |  |  |  |
| WNV knowledge | Between Groups | 64.22 | 2 | 32.11 | 1.21 | 0.31 |
|  | Within Groups | 1165.99 | 44 | 26.50 |  |  |
|  | Total | 1230.21 | 46 |  |  |  |
| Total Sum knowledge | Between Groups | 199.0 | 2 | 99.49 | 0.32 | 0.73 |
|  | Within Groups | 13908.42 | 44 | 316.10 |  |  |
|  | Total | 14107.40 | 46 |  |  |  |
| ***Practices*** |  |  |  |  |  |  |
| Eliminates water | Between Groups | 0.68 | 2 | 0.34 | 0.42 | 0.66 |
|  | Within Groups | 28.16 | 35 | 0.81 |  |  |
|  | Total | 28.84 | 37 |  |  |  |
| Uses repellent | Between Groups | 2.57 | 2 | 1.28 | 0.83 | 0.44 |
|  | Within Groups | 66.30 | 43 | 1.54 |  |  |
|  | Total | 68.87 | 45 |  |  |  |
| Uses insecticide | Between Groups | 7.54 | 2 | 3.77 | 1.82 | 0.18 |
|  | Within Groups | 84.89 | 41 | 2.07 |  |  |
|  | Total | 92.43 | 43 |  |  |  |
| Uses BTI | Between Groups | 5.76 | 2 | 2.88 | 1.86 | 0.17 |
|  | Within Groups | 60.36 | 39 | 1.55 |  |  |
|  | Total | 66.12 | 41 |  |  |  |
| Wears protective clothing | Between Groups | 0.36 | 2 | 0.18 | 1.10 | 0.91 |
|  | Within Groups | 77.95 | 42 | 1.86 |  |  |
|  | Total | 78.31 | 44 |  |  |  |

| Supplemental Table 3. Independent *t*-tests with non-significant results (2-tailed) of ethnicity with knowledge and attitudes. | | | | |
| --- | --- | --- | --- | --- |
| KAP Parameter | Ethnicity | Mean(SD) | df | *p*-value |
| ***Knowledge*** |  |  |  |  |
| General mosquito knowledge | White/Non-Hispanic | 9.78 (1.80) | 43 | **0.018** |
|  | Hispanic | 8.11 (2.74) |  |  |
| DENV knowledge | White/Non-Hispanic | 7.48 (4.31) | 43 | 0.085 |
|  | Hispanic | 5.17 (4.30) |  |  |
| ZIKV knowledge | White/Non-Hispanic | 9.07 (3.91) | 43 | 0.190 |
|  | Hispanic | 7.39 (4.50) |  |  |
| WNV knowledge | White/Non-Hispanic | 9.0 (4.10) | 25.44 | 0.144* |
|  | Hispanic | 11.67 (6.72) |  |  |
| Total sum knowledge | White/Non-Hispanic | 39.11 (13.89) | 25.90 | 0.357* |
|  | Hispanic | 44.61 (22.17) |  |  |
| ***Attitudes*** |  |  |  |  |
| Mosquitoes are a risk | White/Non-Hispanic | 3.11 (0.91) | 43 | 0.440 |
|  | Hispanic | 3.31 (0.65) |  |  |
| MBDs are serious | White/Non-Hispanic | 4.87 (0.39) | 24.63 | 0.076* |
|  | Hispanic | 4.54 (0.68) |  |  |
| Fear of mosquitoes | White/Non-Hispanic | 2.31 (1.01) | 43 | 0.394 |
|  | Hispanic | 2.60 (1.23) |  |  |
| Fear of MBDs | White/Non-Hispanic | 2.56 (1.44) | 43 | **0.010** |
|  | Hispanic | 3.74 (1.48) |  |  |
| Yard maintenance is important | White/Non-Hispanic | 3.94 (0.57) | 43 | 0.684 |
|  | Hispanic | 4.01 (0.58) |  |  |
| City is doing a good job | White/Non-Hispanic | 2.77 (1.08) | 43 | 0.679 |
|  | Hispanic | 2.91 (1.14) |  |  |

*Levene’s test for equality of variance showed that equal variance cannot be assumed

| Supplemental Table 4. Global multiple regressions for attitudes predicting practices | | | | | |
| --- | --- | --- | --- | --- | --- |
| **Practice** | **Predictors (- or + association)** | ***df*** | **R^2^** | ***F*** | ***p*** |
| Uses Insecticides | *Attitudes* |  |  |  |  |
|  | Mosquitoes are a risk (+)  MBDs are serious (+)  City is doing a good job (-)  Yard maintenance is important (+)  Fear of MBDs (-)  Fear of Mosquitoes (+) | 6,40 | 0.239 | 2.094 | 0.075 |
| Eliminates Water | *Attitudes* |  |  |  |  |
|  | Mosquitoes are a risk (+)  MBDs are serious (+)  City is doing a good job (+)*  Yard maintenance is important (+)  Fear of MBDs (+)  Fear of Mosquitoes (-)* | 6,34 | 0.343 | 2.953 | 0.020 |
| Uses Repellant | *Attitudes* |  |  |  |  |
|  | Mosquitoes are a risk (+)*  MBDs are serious (+)  City is doing a good job (-)  Yard maintenance is important (+)  Fear of MBDs (+)  Fear of Mosquitoes (-)* | 6,42 | 0.384 | 4.368 | 0.002 |
| Wears Protective Clothing | *Attitudes* |  |  |  |  |
|  | Mosquitoes are a risk (+)  MBDs are serious (+)  City is doing a good job (+)  Yard maintenance is important (+)  Fear of MBDs (-)  Fear of Mosquitoes (-) | 6,41 | 0.234 | 2.091 | .075 |
| Avoids Being Outside | *Attitudes* |  |  |  |  |
|  | Mosquitoes are a risk (+)  MBDs are serious (+)  City is doing a good job (-)  Yard maintenance is important (-)  Fear of MBDs(+)  Fear of Mosquitoes (-) | 6,38 | 0.132 | 0.965 | 0.462 |
| Uses BTI Briquettes | *Attitudes* |  |  |  |  |
|  | Mosquitoes are a risk (+)  MBDs are serious (+)  City is doing a good job (-)  Yard maintenance is important (+)  Fear of MBDs (-)  Fear of Mosquitoes (+) | 6,38 | 0.051 | 0.338 | 0.912 |

| Supplemental Table 5. Global multiple regressions for personalities predicting practices | | | | | |
| --- | --- | --- | --- | --- | --- |
| **Practice** | **Predictors (- or + association)** | ***df*** | **R^2^** | ***F*** | ***p*** |
| Uses Insecticides | *Personalities* |  |  |  |  |
|  | Agreeableness (+)  Consciousness (+)  Extraversion (+)  Neuroticism (-)  Openness to Experience (-) | 5,41 | 0.214 | 2.237 | 0.069 |
| Eliminates Water | *Personalities* |  |  |  |  |
|  | Agreeableness (-)  Consciousness (+)  Extraversion (+)  Neuroticism (+)  Openness to Experience (-) | 5,35 | 0.241 | 2.223 | 0.074 |
| Uses Repellant | *Personalities* |  |  |  |  |
|  | Agreeableness (-)  Consciousness (-)  Extraversion (+)  Neuroticism (-)  Openness to Experience (-) | 5,43 | 0.154 | 1.570 | 0.189 |
| Wears Protective Clothing | *Personalities* |  |  |  |  |
|  | Agreeableness (+)  Consciousness (-)  Extraversion (+)  Neuroticism (+)  Openness to Experience (+) | 5,42 | 0.061 | 0.549 | .738 |
| Avoids Being Outside | *Personalities* |  |  |  |  |
|  | Agreeableness (-)  Consciousness (+)  Extraversion (-)  Neuroticism (+)  Openness to Experience (-) | 5,39 | 0.035 | 0.280 | 0.921 |
| Uses BTI Briquettes | *Personalities* |  |  |  |  |
|  | Agreeableness (-)  Consciousness (+)  Extraversion (-)  Neuroticism (-)  Openness to Experience (+) | 5,39 | 0.115 | 1.017 | 0.421 |
