# Supplementary material for: Personality and city culture predict attitudes and practices toward mosquitoes and mosquito-borne diseases in South Texas
Source: Front Public Health. 2022 Nov 7;10:919780. doi: 10.3389/fpubh.2022.919780 (PMC9676665; doi:10.3389/fpubh.2022.919780)
Supplement: Supplementary file 3 [file Data_Sheet_2.docx]

Fight the Bite! - Study 2

Start of Block: Consent form and Attitudes

Q1.2 Please indicate your agreement with the following statements:

|  | Strongly disagree 1 (1) | Somewhat disagree 2 (2) | Neither agree nor disagree 3 (3) | Somewhat agree 4 (4) | Strongly agree 5 (5) |
| --- | --- | --- | --- | --- | --- |
| I have sufficient funds for mosquito repellent. (1) |  |  |  |  |  |
| Mosquito control is important to me. (2) |  |  |  |  |  |
| Yard appearance is important to me. (3) |  |  |  |  |  |
| I would rather keep my kids indoors than go outside. (4) |  |  |  |  |  |
| I have sufficient time for yard maintenance. (5) |  |  |  |  |  |
| I have sufficient funds for yard maintenance. (6) |  |  |  |  |  |
| I need to have a clean yard. (7) |  |  |  |  |  |
| I care about what others think about my lawn. (8) |  |  |  |  |  |

Q1.3 Please indicate your agreement with the following statements:

|  | Strongly disagree 1 (1) | Somewhat disagree 2 (2) | Neither agree nor disagree 3 (3) | Somewhat agree 4 (4) | Strongly agree 5 (5) |
| --- | --- | --- | --- | --- | --- |
| I would rather stay indoors than go outside without mosquito repellent. (1) |  |  |  |  |  |
| I feel comfortable going outside without mosquito repellent. (2) |  |  |  |  |  |
| I am motivated to put on mosquito repellent. (3) |  |  |  |  |  |
| It makes me nervous if I forget to put on mosquito repellent. (4) |  |  |  |  |  |
| I do not mind being bitten by mosquitoes. (5) |  |  |  |  |  |
| There is a mosquito problem in my neighborhood. (6) |  |  |  |  |  |
| There is a mosquito problem at my house. (7) |  |  |  |  |  |
| I worry about mosquitoes every time I am outside. (8) |  |  |  |  |  |

Q1.4 Please indicate your agreement with the following statements:

|  | Strongly disagree 1 (1) | Somewhat disagree 2 (2) | Neither agree nor disagree 3 (3) | Somewhat agree 4 (4) | Strongly agree 5 (5) |
| --- | --- | --- | --- | --- | --- |
| I am afraid of insects. (1) |  |  |  |  |  |
| I am afraid of mosquitoes. (2) |  |  |  |  |  |
| I fear getting bitten by mosquitoes. (3) |  |  |  |  |  |
| I fear getting bitten by insects. (4) |  |  |  |  |  |
| I worry about mosquitoes every time I am outside. (5) |  |  |  |  |  |

Q1.5 Please indicate your agreement with the following statements:

|  | Strongly disagree 1 (1) | Somewhat disagree 2 (2) | Neither agree nor disagree 3 (3) | Somewhat agree 4 (4) | Strongly agree 5 (5) |
| --- | --- | --- | --- | --- | --- |
| Zika is a serious disease. (1) |  |  |  |  |  |
| Dengue fever is a serious disease. (2) |  |  |  |  |  |
| West Nile Virus is a serious disease. (3) |  |  |  |  |  |
| Mosquitoes can transmit diseases. (4) |  |  |  |  |  |

Q1.6 Please indicate your agreement with the following statements:

|  | Strongly disagree 1 (1) | Somewhat disagree 2 (2) | Neither agree nor disagree 3 (3) | Somewhat agree 4 (4) | Strongly agree 5 (5) |
| --- | --- | --- | --- | --- | --- |
| The city of San Antonio provides information about mosquitoes. (1) |  |  |  |  |  |
| The city of San Antonio provides enough information about West Nile Virus. (2) |  |  |  |  |  |
| The city of San Antonio provides enough information about Dengue Fever. (3) |  |  |  |  |  |
| The city of San Antonio provide enough information about Zika. (4) |  |  |  |  |  |
| The city of San Antonio does a good job of controlling/preventing mosquitoes. (5) |  |  |  |  |  |

Q1.7 Please indicate your agreement with the following statements:

|  | Strongly disagree 1 (1) | Somewhat disagree 2 (2) | Neither agree nor disagree 3 (3) | Somewhat agree 4 (4) | Strongly agree 5 (5) |
| --- | --- | --- | --- | --- | --- |
| I am afraid of getting West Nile virus. (1) |  |  |  |  |  |
| I am afraid of getting the Zika virus. (2) |  |  |  |  |  |
| I am afraid of getting Dengue fever. (3) |  |  |  |  |  |

End of Block: Consent form and Attitudes

Start of Block: NEO IPIP

Q2.1 Please indicate your agreement with the following statements:

|  | Strongly disagree 1 (1) | Somewhat disagree 2 (2) | Neither agree nor disagree 3 (3) | Somewhat agree 4 (4) | Strongly agree 5 (5) |
| --- | --- | --- | --- | --- | --- |
| I am the life of the party. (1) |  |  |  |  |  |
| I sympathize with others’ feelings (2) |  |  |  |  |  |
| I Get chores done right away. (3) |  |  |  |  |  |
| I have frequent mood swings. (4) |  |  |  |  |  |
| I have a vivid imagination. (5) |  |  |  |  |  |
| I don’t talk a lot. (6) |  |  |  |  |  |
| I am not interested in other people’s problems. (7) |  |  |  |  |  |
| I often forget to put things back in their proper place. (8) |  |  |  |  |  |
| I am relaxed most of the time. (9) |  |  |  |  |  |
| I am not interested in abstract ideas. (10) |  |  |  |  |  |
| I talk to a lot of different people at parties. (11) |  |  |  |  |  |
| I feel others’ emotions. (12) |  |  |  |  |  |
| I like order. (13) |  |  |  |  |  |
| I get upset easily. (14) |  |  |  |  |  |
| I have difficulty understanding abstract ideas. (15) |  |  |  |  |  |
| I keep in the background. (16) |  |  |  |  |  |
| I am not really interested in others. (17) |  |  |  |  |  |
| I make a mess of things. (18) |  |  |  |  |  |
| I seldom feel blue. (19) |  |  |  |  |  |
| I do not have a good imagination. (20) |  |  |  |  |  |

End of Block: NEO IPIP

Start of Block: Knowledge

Q3.1 Have you ever heard of West Nile virus?

- Yes (1)
- No (2)

Skip To: Q3.9 If Have you ever heard of West Nile virus? = No

Q3.2 Please answer the following questions:

|  | Yes (1) | No (2) | Don't know (3) |
| --- | --- | --- | --- |
| Based on what you know, do you think that people can get infected with the West Nile Virus? (3) |  |  |  |
| Do you think that animals can get infected with West Nile virus? (4) |  |  |  |

Skip To: Q3.4 If Please answer the following questions: = Do you think that animals can get infected with West Nile virus? [ No ]

Skip To: Q3.4 If Please answer the following questions: = Do you think that animals can get infected with West Nile virus? [ Don't know ]

Q3.3 Which animal(s), if any, can be infected with West Nile virus (select all that apply):

- Birds (1)
- Dogs (2)
- Cats (3)
- Horses (4)
- Cows (5)
- Chickens (6)
- Bats (7)
- Squirrels (8)
- Rats (9)
- Other Please specify: (11) __________________________________________________
- Don't know (12)

Q165 Please answer the following question:

|  | Yes (1) | No (2) | Yes, but only old & young (3) |
| --- | --- | --- | --- |
| Can West Nile virus kill you? (1) |  |  |  |

Q166 Please answer the following question:

|  | Yes (1) | No (2) |
| --- | --- | --- |
| Does everyone who gets West Nile virus show symptoms? (1) |  |  |

Q3.4 If someone were infected with West Nile virus, how would they know? That is, what symptoms do you think they would have? (select all that apply)

- Fever (2)
- Rash (9)
- Headache (3)
- Tiredness (4)
- Joint Pain (10)
- Neck stiffness (5)
- Nausea (6)
- Convulsions (7)
- Conjunctivitis (red eyes) (11)
- Muscle pain or weakness (12)
- Other Please specify: (13) __________________________________________________
- Don't know (14)

Q3.5 Who do you think is **most** at risk for developing disease caused by West Nile virus? (select all that apply)

- Everyone (1)
- Babies still in their mother’s womb (6)
- Baby/toddlers (2)
- Children (3)
- Adults (4)
- Elderly >55 (5)
- Other Please specify: (7) __________________________________________________
- Don't know (8)

Q3.6 How do people get infected with West Nile virus? (Select all that apply)

- Bite of an infected mosquito (1)
- Other insects/ticks (2)
- From another human that is infected (3)
- Touching infected animals (4)
- Touching infected birds (5)
- Blood transfusion (6)
- Other Please specify (7) __________________________________________________
- Don't know (8)

Q3.7 When was the first time you heard of West Nile virus? Provide approximate year (e.g. 2006)

________________________________________________________________

Q3.8 If someone were infected with West Nile virus, what do you think is the chance of that person getting sick? Give percentage %

________________________________________________________________

Q3.9 Have you ever heard of Zika?

- Yes (1)
- No (2)

Skip To: Q3.17 If Have you ever heard of Zika? = No

Q3.10 Please answer the following questions:

|  | Yes (1) | No (2) | Don't know (3) |
| --- | --- | --- | --- |
| Based on what you know, do you think that people can get infected with Zika? (2) |  |  |  |
| Do you think that animals can get infected with Zika? (3) |  |  |  |
| Does everyone who gets Zika virus show symptoms? (1) |  |  |  |

Q3.12 If someone were infected with Zika, how would they know? That is, what symptoms do you think they would have? (Select all that apply)

- Fever (2)
- Rash (9)
- Headache (3)
- Tiredness (4)
- Joint Pain (10)
- Neck stiffness (5)
- Nausea (6)
- Convulsions (7)
- Conjunctivitis (red eyes) (11)
- Muscle pain or weakness (12)
- Other Please specify: (13) __________________________________________________
- Don't know (14)

Q3.12 Serious complications associated with Zika are (Select all that apply):

- Development of Guillain-Barre Syndrome (1)
- Microcephaly (extremely small head of newborn baby) (2)
- Other birth defects (3)
- Death if infected as an adult (4)
- Death associated with birth defects (5)
- Other Please Specify: (6) __________________________________________________
- Don't know (7)

Q3.13 Who do you think is most at risk for developing serious complications associated with Zika?

- Everyone (1)
- Babies while still in mother’s womb (6)
- Baby/toddlers (2)
- Children (3)
- Adults (4)
- Elderly >55 (5)
- Other Please Specify: (7) __________________________________________________
- Don't know (8)

Q3.14 How do people get infected with Zika? (Select all that apply)

- Bite of an infected mosquito (1)
- Through sexual intercourse (7)
- From mother to unborn baby (8)
- Other insects/ticks (2)
- Touching infected animals (4)
- Touching infected birds (5)
- Blood transfusion (6)
- Other Please Specify: (9) __________________________________________________
- Don't know (10)

Q3.15 When was the first time you heard of Zika? Provide year (e.g. 2006)

________________________________________________________________

Q3.16 If someone were infected with Zika, what do you think is the chance of that person getting sick? Give percentage %

________________________________________________________________

Q3.17 Have you ever heard of Dengue fever?

- Yes (1)
- No (2)

Skip To: End of Block If Have you ever heard of Dengue fever? = No

Q3.18 Please answer the following questions:

|  | Yes (1) | No (2) | Don't know (3) |
| --- | --- | --- | --- |
| Based on what you know, do you think that people can get infected with Dengue fever? (3) |  |  |  |
| Do you think that animals can get infected with Dengue fever? (4) |  |  |  |
| Does everyone who gets Dengue fever show symptoms? (2) |  |  |  |
| Can Dengue fever kill you? (1) |  |  |  |

Q3.20 If someone were infected with Dengue fever, how would they know? That is, what symptoms do you think they would have? (Select all that apply)

- Severe headache (3)
- Severe eye pain (behind eyes) (9)
- Joint pain, muscle, and or bone pain (10)
- Rash (11)
- Mild bleeding manifestation (e.g., nose or gum bleed, petechiae, or easy bruising (12)
- Convulsions (7)
- Conjunctivitis (red eyes) (13)
- Other Please Specify: (14) __________________________________________________
- Don't know (15)

Q3.20 Serious complications associated with Dengue Fever are (Select all that apply):

- Development of Dengue Hemorraghic Fever (1)
- Birth defects (2)
- Death (3)
- Other Please Specify: (4) __________________________________________________
- Don't know (5)

Q3.21 Who do you think is **most** at risk for developing disease caused by Dengue fever?

- Everyone (1)
- Babies while still in mother’s womb (6)
- Baby/toddlers (2)
- Children (3)
- Adults (4)
- Elderly >55 (5)
- Those who have had previous infections with Dengue (7)
- Other Please Specify: (8) __________________________________________________
- Don't know (9)

Q3.22 How do people get infected with Dengue fever? (Select all that apply)

- Bite of an infected mosquito (1)
- Through sexual intercourse (7)
- From mother to unborn baby (8)
- Other insects/ticks (2)
- Touching infected animals (4)
- Touching infected birds (5)
- Blood transfusion (6)
- Other Please Specify: (9) __________________________________________________
- Don't know (10)

Q3.23 When was the first time you heard of Dengue fever? Provide year (e.g. 2006)

________________________________________________________________

Q3.24 If someone were infected with Dengue fever, what do you think is the chance of that person getting sick? Give percentage %

________________________________________________________________

End of Block: Knowledge

Start of Block: General Knowledge

Q4.1 Does the species of mosquito that carries West Nile virus live in your area?

- Yes (1)
- No (2)
- Don't know (3)

Q4.2 Does the species of mosquito that carries Zika live in your area?

- Yes (1)
- No (2)
- Don't know (3)

Q4.3 Does the species of mosquito that carries Dengue fever live in your area?

- Yes (1)
- No (2)
- Don't know (3)

Q4.4 Who do you trust most to give you information about diseases that you can get from a mosquito bite? (Select all that apply)

- Friend (1)
- Family member (2)
- Doctor or physician (3)
- Radio (4)
- T.V. (5)
- Newspaper (6)
- Internet (7)
- The city of San Antonio Metro Health (8)
- Other Please specify: (9) __________________________________________________
- Don't know (10)

Q4.5 What can people do to reduce the risk of becoming infected with viruses mosquitoes can transmit to humans? (Select all that apply)

- Insect repellent (1)
- Protect skin with clothes (2)
- Staying indoors at dawn, dusk and early evening (3)
- Citronella candles (4)
- Avoid outdoor areas with mosquitoes (5)
- Screening of windows (6)
- Eliminate sources of standing water (7)
- Pesticide sprays (8)
- Vaccination (9)
- Other Please specify: (10) __________________________________________________
- Don't know (11)

Q4.6 What are important breeding places for mosquitoes?

- Containers in yard with standing water (1)
- Swamp (2)
- Brush and Vegetation (3)
- Swimming pools (4)
- Other Please Specify: (5) __________________________________________________
- Don't know (6)

End of Block: General Knowledge

Start of Block: Practices

Q5.1 How would you describe the level of mosquito activity near your home?

- Low (1)
- Medium (2)
- High (3)

Q5.2 Do you have screening on your windows?

- Yes (1)
- No (2)
- Partially (3)
- Don’t know (4)

Skip To: Q5.4 If Do you have screening on your windows? = Yes

Skip To: Q5.4 If Do you have screening on your windows? = Yes

Q5.3 What are the main reasons for not having screening on your windows?

________________________________________________________________

Q5.4 When you’re outside during mosquito season, do you wear protective clothing (long-sleeved shirts and long pants)?

- Never (1)
- Occasionally (2)
- Half of the time (3)
- Often (4)
- Always (5)
- Other (6) __________________________________________________
- Don't know (7)

Skip To: Q5.11 If When you’re outside during mosquito season, do you wear protective clothing (long-sleeved shirts... = Always

Skip To: Q5.5 If When you’re outside during mosquito season, do you wear protective clothing (long-sleeved shirts... = Never

Skip To: Q5.6 If When you’re outside during mosquito season, do you wear protective clothing (long-sleeved shirts... = Always

Skip To: Q5.6 If When you’re outside during mosquito season, do you wear protective clothing (long-sleeved shirts... = Don't know

Q5.5 What are the main reasons for not wearing protective clothing when you’re outside during mosquito season?

________________________________________________________________

Q5.6 How many evenings a week do you stay outside more than 30 minutes?  Please specify.

- Evenings per week (1) __________________________________________________
- Don't know (2)

Q5.7 Do you avoid being outside during mosquito season?

- Never (1)
- Occasionally (2)
- Half of the time (3)
- Often (4)
- Always (5)
- Don't know (6)

Q5.8 Which of the following, if any, do you currently have in your yard?

- Bird bath or unused fountain (1)
- uncovered rain barrel (2)
- If own a pool - pool cover (3)
- pots/cups/buckets (4)
- discarded tires (including swings) (5)
- children's toys (6)
- decorative pond without fish (7)
- uncovered trash bins (8)
- pet bowls (9)

Q5.9 How often do you eliminate standing water from any of the items in the previous question?

- Never (1)
- At least once a week (2)
- About every other week (3)
- Once a month (4)
- Don't know (5)

Skip To: Q5.11 If How often do you eliminate standing water from any of the items in the previous question? = Never

Skip To: Q5.11 If How often do you eliminate standing water from any of the items in the previous question? = At least once a week

Skip To: Q5.11 If How often do you eliminate standing water from any of the items in the previous question? = Don't know

Q5.10 What are the main reasons for not eliminating standing water from those containers?

________________________________________________________________

Q5.11 Do you use insect repellent?

- Never (1)
- Occasionally (2)
- Half of the time (3)
- Often (4)
- Always (5)
- Don't know (6)

Skip To: Q5.13 If Do you use insect repellent? = Never

Skip To: Q5.12 If Do you use insect repellent? = Never

Skip To: Q5.14 If Do you use insect repellent? = Don't know

Q5.12 What is the active ingredient in your repellent?

- DEET (1)
- Picaridin (2)
- Oil of lemon Eucalyptus (p-menthane 3,8-diol (PMD)) (3)
- Citronella (4)
- Permethrin (5)
- Other (6) __________________________________________________
- Don't know (7)

Q5.13 Do you have any concerns about using insect repellents? If yes, please explain why:

- Yes (1) __________________________________________________
- No (2)
- Don't know (3)

Q5.14 Which of the following do you use to prevent mosquitoes from biting you or a member of your family?

|  | Never (1) | Occasionally (2) | Half the time (3) | Often (4) | Always (5) | Don't know (6) |
| --- | --- | --- | --- | --- | --- | --- |
| Ultrasonic devices (1) |  |  |  |  |  |  |
| Electronic mosquito killers (2) |  |  |  |  |  |  |
| Mosquito magnets (3) |  |  |  |  |  |  |
| Fans (4) |  |  |  |  |  |  |
| Citronella candles (5) |  |  |  |  |  |  |
| B.t.i briquets or pellets (6) |  |  |  |  |  |  |
| Bed nets (7) |  |  |  |  |  |  |
| Insecticides (8) |  |  |  |  |  |  |
| Vicks Vapor rub (9) |  |  |  |  |  |  |
| Incense (10) |  |  |  |  |  |  |
| Garlic (11) |  |  |  |  |  |  |
| Coconut oil (12) |  |  |  |  |  |  |
| Essential oils (13) |  |  |  |  |  |  |
| Listerine (14) |  |  |  |  |  |  |

End of Block: Practices

Start of Block: Demographics

Q6.1 What is your age?

________________________________________________________________

Q6.2 Please select your ethnicity:

- White (1)
- Black or African American (2)
- American Indian or Alaskan Native (3)
- Asian (4)
- Hispanic (5)
- Native Hawaiian or Other Pacific Islander (6)
- Other Please specify: (7) __________________________________________________

Q6.3 What is your gender?

- Male (1)
- Female (2)
- Other (3)

Q6.4 How many people are in your household? (e.g. 6)

________________________________________________________________

Q6.5 How many people in your household are under 18? (e.g. 2)

________________________________________________________________

Q6.6
How many people in your household are over 65? (e.g. 2)

________________________________________________________________

Q6.7 What is your annual household income?

- $0-$14,999 (1)
- $15,000 - $49,999 (2)
- $50,000-$74,999 (3)
- $75,000-$99,999 (4)
- $100,000- $124,999 (5)
- $125,000-$149,999 (6)
- $150,000-$174,999 (7)
- $175,000-$199,999 (8)
- $200,000 and up (9)

Q170 What is your highest level of education?

- Did not finish high school (1)
- High School Diploma (2)
- Some College (3)
- Associate's Degree (4)
- Bachelor's Degree (5)
- Some graduate school (6)
- Master's degree, law degree, or other graduate technical degree (7)
- Doctoral degree (8)

Q6.10 Does anyone in your household have an outdoor job?

- Yes (1)
- No (2)

Q6.11 Do you spend at least half of your time outdoors at your job?

- Yes (1)
- No (2)

Q6.12 Do you treat your yard for mosquitoes regularly?

- Yes (1)
- No (2)

Q6.13
Have you been pregnant any time during the years 2015-2018?

- Yes (1)
- No (2)

Q6.14 Do you travel outside of the U.S. at least twice a year? If so, which country(ies)?

________________________________________________________________

Q6.15 Do you travel within the U.S at least twice a year? If so, which state(s)?  

________________________________________________________________

Q6.16 What is your address (so the researcher does not visit your house)?

________________________________________________________________
